# Supplementary material for: Nivolumab plus chemoradiotherapy in locally-advanced cervical cancer: the NICOL phase 1 trial
Source: Nat Commun. 2023 Jun 22;14:3698. doi: 10.1038/s41467-023-39383-8 (PMC10287640; doi:10.1038/s41467-023-39383-8)
Supplement: Supplementary file 1 — Supplementary Information [file 41467_2023_39383_MOESM1_ESM.pdf]

Supplementary Information for

Nivolumab plus chemoradiotherapy in locally-advanced cervical cancer: the NICOL phase 1 trial

Manuel Rodrigues[1*], Giulia Vanoni[2*], Pierre Loap[3], Coraline Dubot[1], Eleonora Timperi[2], Mathieu Minsat[3], Louis Bazire[3], Catherine Durdux[4], Virginie Fourchotte[5], Enora Laas[5], Nicolas Pouget[5], Zahra Castel-Ajgal[6], Gregoire Marret[6], Laetitia Lesage[7,8], Didier Meseure[7,8], Anne Vincent-Salomon[7,8], Lolita Lecompte[9], Nicolas Servant[9], Sophie Vacher[10], Ivan Bieche[10], Caroline Malhaire[11], Virginie Huchet[12], Laurence Champion[12], Maud Kamal[6], Sebastian Amigorena[2], Olivier Lantz[2], Marion Chevrier[13], Emanuela Romano[1,2§]

Affiliations:
[1] Department of Medical Oncology, Institut Curie, Paris & Saint-Cloud, France;
[2] Center for Cancer Immunotherapy, INSERM U932, PSL Research University, Institut Curie;
[3] Department of Radiation Oncology, Institut Curie, Paris & Saint Cloud, France;
[4] Hôpital Européen Georges Pompidou, Department of Radiation Oncology, Paris, France;
[5] Service of Breast and Gynecologic Surgery, Institut Curie, Paris France;
[6] Department of Drug Development and Innovation, Institut Curie, Paris, France;
[7] Department of Pathology Institut Curie, Paris, France;
[8] Centre d'Investigation Clinique Biothérapie, Institut Curie, Paris, France;
[9] Institut Curie Bioinformatics Platform, INSERM U900, Mines ParisTech ; Paris 75005, France;
[10] Pharmacogenomics Unit, Service of Genetics, Institut Curie, Paris, France ;
[11] Department of Radiology, Institut Curie, Paris 75005, France;
[12] Department of Nuclear Medicine, Institut Curie, Paris 75005, France;
[13] Service of Biostatistics, Institut Curie, Paris 75005, France.

* These authors contributed equally

[§]Corresponding Author:
Dr Emanuela Romano
Institut Curie
26, rue d'Ulm
Paris 75005, France
Emanuela.Romano@curie.fr

This PDF file includes:

Supplementary Figures 1, 2, 3, 4
Supplementary Tables 1, 2, 3
Supplementary Note (Study Protocol)

Supplementary Figure 1

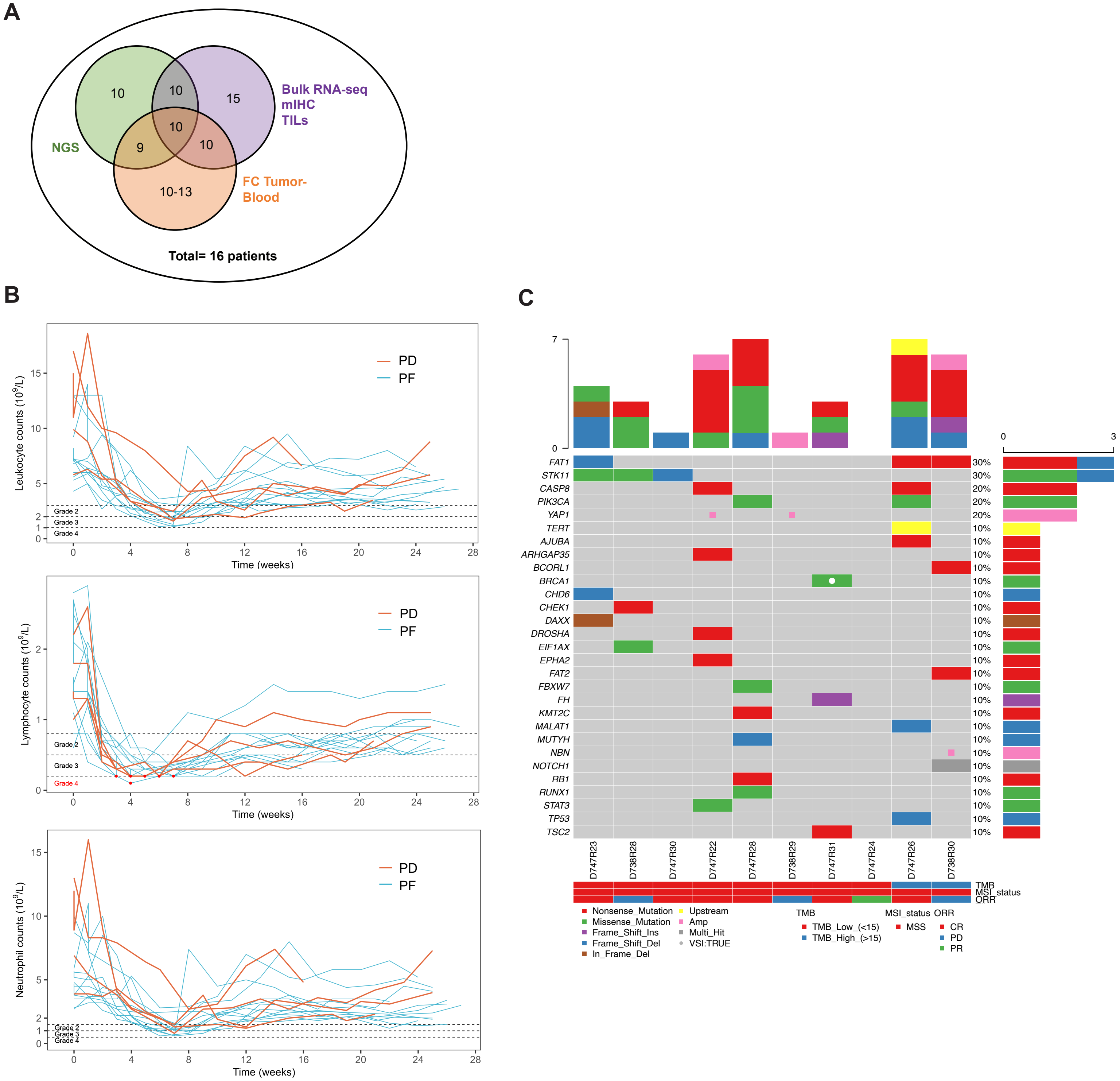

Supplementary Figure 1. Nicol patient cohort and OncoPrint. (**A**) Venn diagram showing the overlap of patients across the different collected datasets. A total of 16 patients were included in the clinical trial. TILs assessment, mIHC and Bulk RNA-seq were performed for 15/16 patients at baseline, comprising 11PF and 4PD patients. NGS was performed for all patients, but only 10/16 were of good quality, comprising 7PF (6CR+1PR) and 3PD. FC analyses were performed for 10/16 baseline tumors (comprising 6PF and 4PD patients). FC analyses of PBMCs were performed at baseline for 10/16 patients (n=7 for PF, n=3 for PD), at week 3 and week 6 for 13/16 patients (n=9 for PF, n=4 for PD). (**B**) Spaghetti plots of peripheral Leukocyte (top), Lymphocyte (middle) and Neutrophil (bottom) counts fluctuation over time in patients with progressive disease (orange lines) and progression-free (light blue lines). The black dotted lines indicate the grade of leukopenia, lymphopenia, and neutropenia, respectively, according to the CTCAE version 4.03. (**C**) OncoPrint plot of 10 baseline tumors (comprising 7PF (6CR+1PR) and 3PD) showing the most frequently altered genes. This figure provides an overview of the most frequent genomic alterations (left column) with their respective frequencies (right column) combined with clinical information, Microsatellite instability (MSI) and tumor mutational burden (TMB) data (heading). Each column represents a patient. Each type of genomic alteration is represented by a color-code. Panel B: n=12 for PF (comprising 11 CR and 1 PR), n=4 for PD; Panel C: n=7 for PF (comprising 6 CR and 1 PR), n=3 for PD (biologically independent samples). MSS= Micro Satellite Stable; ORR= Overall Response Rate; CR=Complete Response; PD=Progressive Disease; PR= Partial Response. NGS= Next Generation Sequencing; mIHC = multiplexed immunohistochemistry; TILs= tumor-infiltrating lymphocytes. FC= flow cytometry. Source data relative to panel B are provided as Source Data File.

Supplementary Figure 2

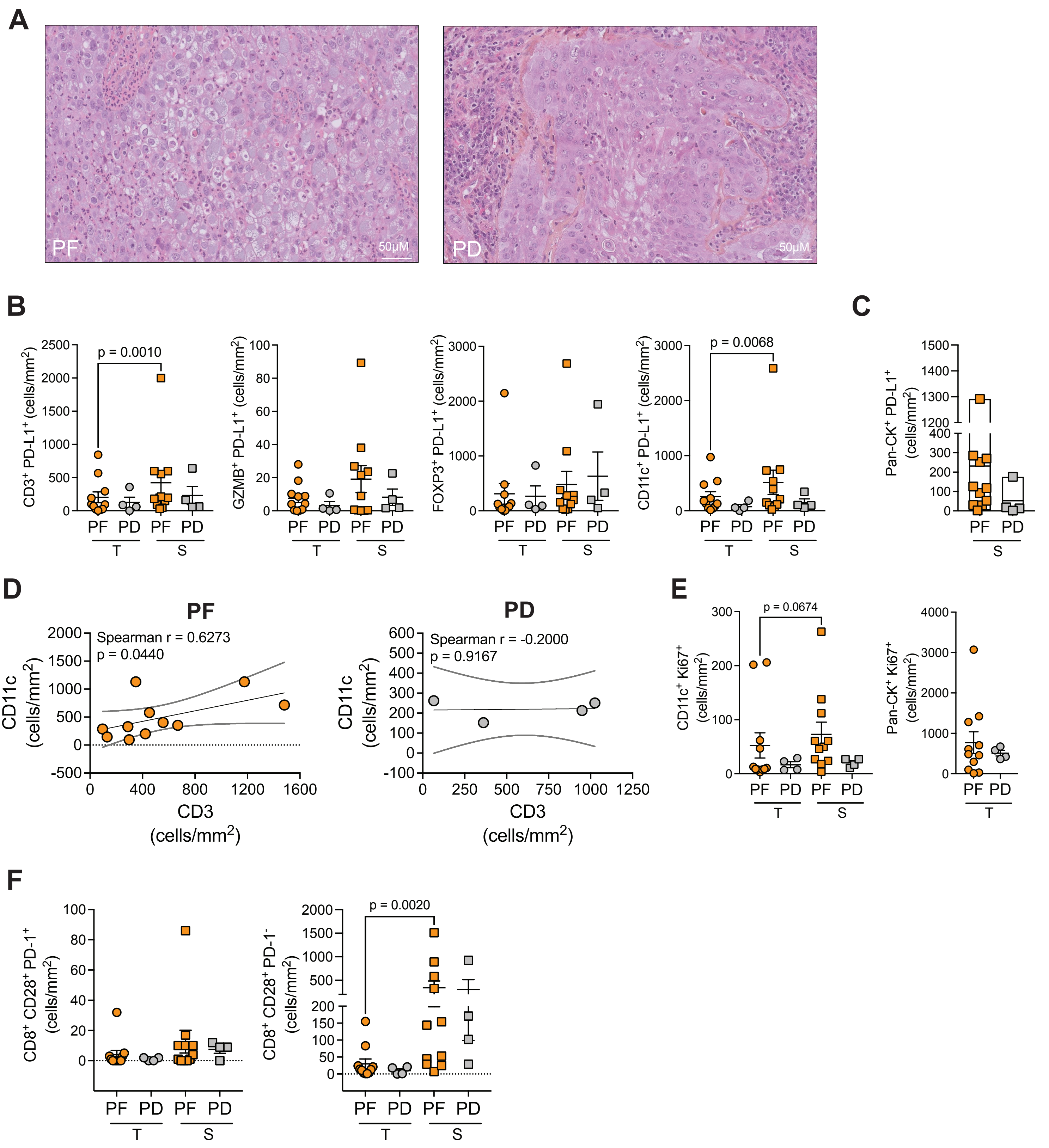

Supplementary Figure 2: Profiling of intratumoral T and myeloid cells. (**A**) Representative H&E staining of tumor-infiltrating lymphocytes (TILs) in the tumor area of a PF (on the left) and of a PD (on the right) (20x magnification). (**B**) Number of $CD3^+$, $GZMB^+$, $FOXP3^+$ and $CD11c^+$ cells co-expressing PD-L1 in the stroma and tumor areas, expressed as cells per mm^2, in PF *vs* PD. (**C**) Number of Pan-CK^+ PD-$L1^+$ cells in the invasive margin (stroma). (**D**) Spearman correlation between the number of $CD11c^+$ and $CD3^+$ positive cells in the T area, assessed by mIF, in PF *vs* PD (cells/mm^2). The graphs display non-linear regression curves as well as 95% confidence intervals. (**E**) Number of proliferating ($Ki67^+$) $CD11c^+$ cells in the S *vs* T areas and of Pan-CK^+ tumor cells in the T area, expressed as cells per mm^2, in PF *vs* PD. (**F**) Number of $CD8^+CD28^+PD\text{-}1^+$ (left) and $CD8^+CD28^+PD\text{-}1^-$ (right) cells in the S *vs* T areas, expressed as cells per mm^2, in PF *vs* PD. In all panels, PF: n=11; PD: n=4 (biologically independent samples). Panel A is representative of 15 biologically independent samples. Data are presented as individual values showing mean ± SEM. Statistical tests: two-tailed Wilcoxon matched-pairs signed rank test (Panels B-C, E-F); non-parametric Spearman correlation (Panel D). If not indicated, no statistically significant difference was observed. T=Tumor; S = Stroma. Source data relative to panels B-F are provided as Source Data File.

Supplementary Figure 3

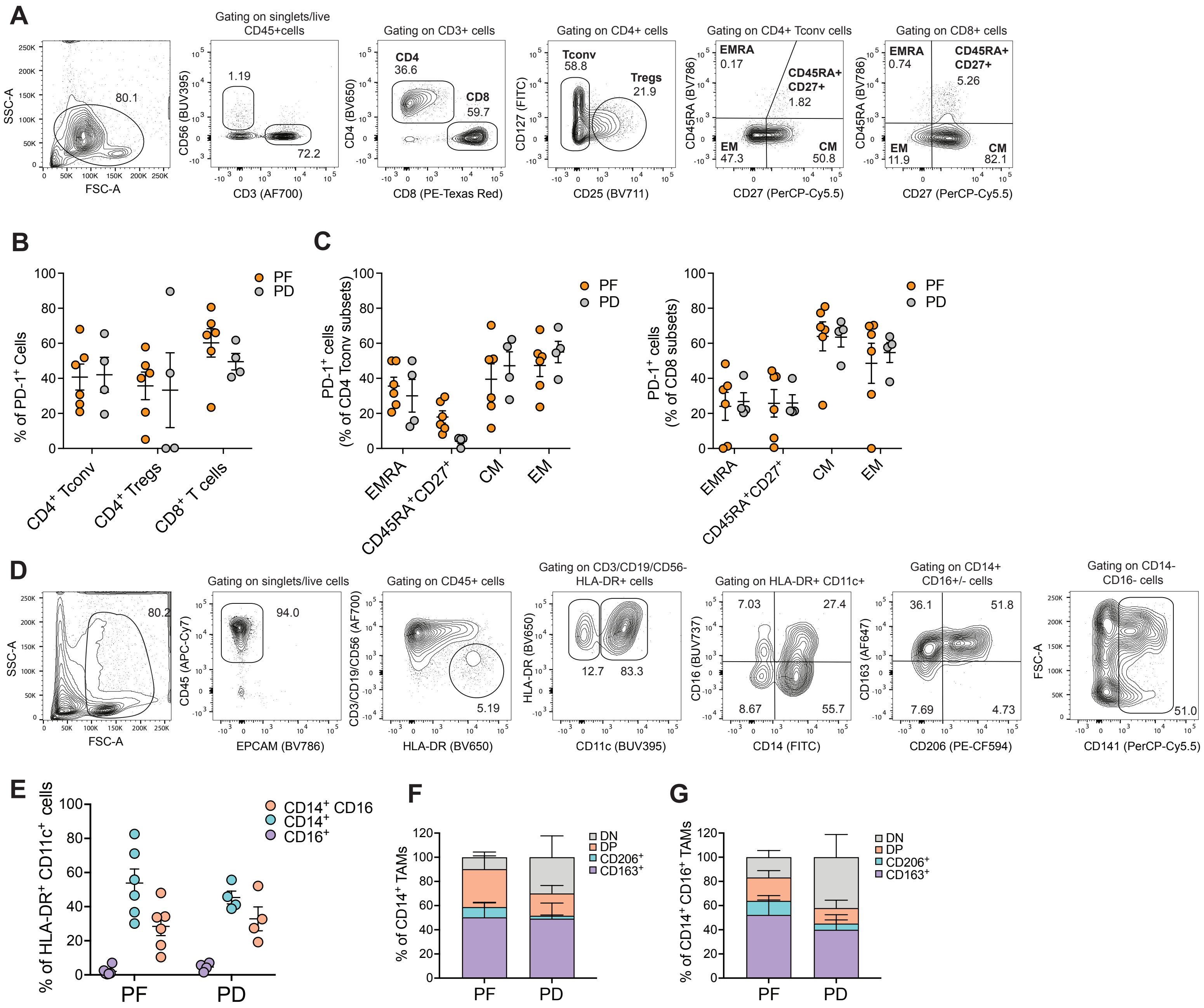

Supplementary Figure 3: Gating strategy, phenotype, and distribution of intra-tumoral T and myeloid cell subsets at baseline. (**A**) Gating strategy for the identification of intra-tumoral CD3$^+$ T-cell subpopulations by FC (representative plots from a PD). Gating on singlets, Aqua Live/Dead$^-$ CD45$^+$ CD3$^+$ (CD56$^-$) cells, Tconv are defined as CD4$^+$CD127$^{-/+}$CD25$^-$ and Tregs as CD4$^+$CD127^{low}CD25$^{+/high}$. CD45RA and CD27 markers distinguish CD4$^+$ Tconv and CD8$^+$ T cell subpopulations: EM= CD45RA$^-$ CD27$^-$; CM = CD45RA$^-$ CD27$^+$; CD45RA$^+$CD27$^+$ cells; EMRA = CD45RA$^+$CD27$^-$. (**B**) Frequency of PD-1$^+$ cells among CD4$^+$ Tconv, CD4$^+$ Tregs and CD8$^+$ T cells. (**C**) Frequency of PD-1$^+$ cells among CD4$^+$ Tconv (left) and CD8$^+$ T cell subsets (right) (EMRA, CM, CD45RA$^+$CD27$^+$, EM). (**D**) Gating strategy for the visualization of intra-tumoral myeloid lineage subsets by FC (representative plots from a PD). Gating on singlets, Aqua Live/Dead$^-$ CD45$^+$ EPCAM$^-$ Lineage$^-$ (CD3$^-$CD19$^-$CD56$^-$) cells, the total myeloid compartment is defined as HLA-DR$^+$. Then, within HLA-DR$^+$ CD11c$^+$ cells, TAM subsets are identified, according to the expression of CD14$^+$ and CD16$^+$. Within CD14$^+$CD16$^-$ and CD14$^+$CD16$^+$ TAMs, the expression of CD163 and CD206 is analysed. Gating on Lineage$^-$HLA-DR$^+$CD11c$^+$ CD14$^-$CD16$^-$ cells, CD141$^+$ cDC1 cells are visualized. (**E**) Proportions of CD14$^+$, CD16$^+$ and CD14/CD16 double positive TAMs, expressed as frequency of Lineage$^-$HLA-DR$^+$CD11c$^+$ cells. (**F**) Frequency of CD163$^+$, CD206$^+$, double positive and double negative CD14$^+$ TAMs. (**G**) Frequency of CD163$^+$, CD206$^+$, double positive and double negative CD14$^+$CD16$^+$ TAMs. In all panels, PF: n=11, PD: n=4 (biologically independent samples). Data are presented as individual values showing mean ± SEM in PF (orange) and PD (grey). Statistical tests: two-tailed unpaired *t*-test (Mann-Whitney test) for all panels. If not indicated, no significant difference was observed. Tconv= T conventional; Treg = T regulatory; EM = Effector Memory; CM = Central Memory; EMRA= Effector Memory RA. Source data relative to panels B-C and E-G are provided as Source Data File.

Supplementary Figure 4

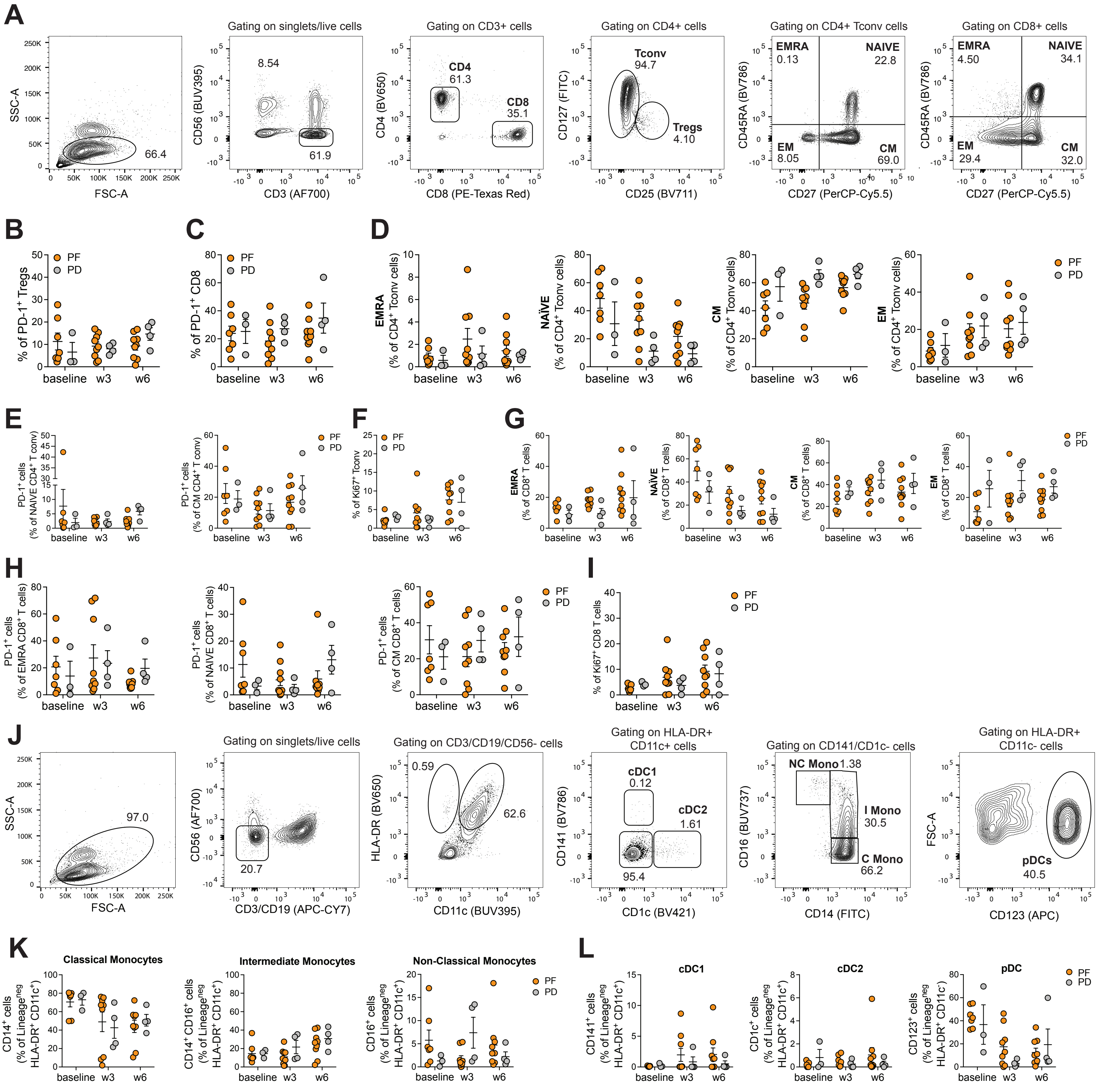

Supplementary Figure 4: Gating strategy, phenotype and distribution of *ex vivo* peripheral T and myeloid cell subsets by flow cytometry. (**A**) Gating strategy for *ex vivo* peripheral T cell subsets. Gating on singlets, Aqua Live/Dead^{-} CD45^{+}CD3^{+} T cells, Tconv are CD4^{+}CD127$^{-/+}$CD25^{-} cells and Tregs are CD4^{+}CD127^{low}CD25$^{+/high}$ cells. CD45RA and CD27 markers distinguish CD4^{+} Tconv and CD8^{+} T cell subpopulations: EM= CD45RA^{-}CD27^{-}; CM = CD45RA^{-}CD27^{+}; NAÏVE= CD45RA^{+}CD27^{+}; EMRA = CD45RA^{+}CD27^{-}. (**B**) Frequency of PD-1^{+} Tregs. (**C**) Frequency of PD-1^{+} cells among CD8^{+} T cells. (**D**) Distribution of CD4^{+} T cell subsets. (**E**) Frequency of PD-1^{+} in NAÏVE (left) and CM (right) CD4^{+} Tconv cells. (**F**) Frequency of Ki67^{+} Tconv. (**G**) Distribution of CD8^{+} T cell subsets. (**H**) Frequency of PD-1^{+} cells in EMRA (left), NAÏVE (center) and CM (right) CD8^{+} T cells. (**I**) Frequency of Ki67^{+} CD8^{+} T cells. (**J**) Gating strategy for *ex* vivo peripheral myeloid lineage cell subsets. Gating on singlets, Aqua Live/Dead- CD45^{+} cells, the total myeloid compartment is defined as Lineage^{-} (CD3^{-}CD19^{-}CD56^{-}) HLA-DR^{+}. pDCs are defined as HLA-DR^{+}CD11c^{+}CD123^{+}. Among HLA-DR^{+}CD11c^{+}CD14^{-}CD16^{-} cells, cDC1 are CD141^{+} cells and cDC2 are CD1c^{+} cells. Within CD141^{-}CD1c^{-} cells, Classical (C) CD14^{+}, Intermediate (I) CD14^{+}CD16^{+}, and Non-Classical (NC) CD16^{+} monocytes are visualized. (**K**) Frequency of monocyte subsets among Lineage^{-}HLA-DR^{+}CD11c^{+}CD141^{-}CD1c^{-} cells. (**L**) Frequency of cDC1s (left), cDC2s (center) and pDCs (right) among Lineage^{-}HLA-DR^{+}CD11c^{+} (for cDC1s and cDC2s) or CD11c^{-} (for pDCs cells). Panels A and J are representative from a PD patient. In all panels, PF baseline: n= 7, PD baseline: n= 3; PF week 3: n= 9, PD week 3: n= 4; PF week 6: n= 9, PD week 6: n= 4 (all biologically independent samples). Data are presented with the mean ± SEM in PF (orange) *vs* PD (grey) at the indicated time points. Statistical test: two-way ANOVA – Mixed-effects model with the Geisser-Greenhouse correction (all panels). Tconv= T conventional; Treg = T regulatory; EM = Effector Memory; CM = Central Memory; EMRA= Effector Memory RA. C Mono= Classical Monocytes. I Mono= Intermediate Monocytes. NC Mono= Non-Classical Monocytes. cDC1= Conventional Dendritic Cell type 1. cDC2= Conventional Dendritic Cell type 2. pDC= plasmacytoid Dendritic Cell. Source data are provided as Source Data File.

Supplementary Table 1: Treatment-related adverse events - worse grade by type of toxicity.

Type of toxicity	Grade 1	Grade 2	Grade 3	Grade 4
	**N (%)**	**N (%)**	**N (%)**	**N (%)**
**Investigations**				
Highest toxicity	-	2 (12.5%)	9 (56.2%)	5 (31.2%)
Alanine aminotransferase increased	4 (25%)	2 (12.5%)	-	-
Aspartate aminotransferase increased	6 (37.5%)	-	-	-
Creatinine increased	1 (6.2%)	1 (6.2%)	-	-
GGT increased	1 (6.2%)	-	-	-
Lymphocyte count decreased	-	1 (6.2%)	8 (50%)	5 (31.2%)
Neutrophil count decreased	2 (12.5%)	4 (25%)	7 (43.8%)	-
Platelet count decreased	7 (43.8%)	3 (18.8%)	-	-
Weight loss	3 (18.8%)	1 (6.2%)	-	-
**Gastrointestinal disorders**				
Highest toxicity	5 (31.2%)	10 (62.5%)	1 (6.2%)	-
Abdominal pain	2 (12.5%)	-	-	-
Constipation	5 (31.2%)	1 (6.2%)	-	-
Diarrhea	7 (43.8%)	4 (25%)	1 (6.2%)	-
Dyspepsia	1 (6.2%)	-	-	-
Gastritis	-	1 (6.2%)	-	-
Gastrointestinal pain	1 (6.2%)	-	-	-
Hemorrhoids	1 (6.2%)	-	-	-
Mucositis oral	3 (18.8%)	-	-	-
Nausea	5 (31.2%)	10 (62.5%)	-	-
Proctitis	-	1 (6.2%)	-	-
Rectal mucositis	1 (6.2%)	-	-	-
Stomach pain	3 (18.8%)	-	-	-
Vomiting	5 (31.2%)	1 (6.2%)	-	-
**General disorders and administration site conditions**				
Highest toxicity	4 (26.7%)	11 (73.3%)	-	-
Chills	1 (6.7%)	1 (6.7%)	-	-
Fatigue	4 (26.7%)	11 (73.3%)	-	-
Fever	-	2 (13.3%)	-	-
**Blood and lymphatic system disorders**				
Highest toxicity	3 (21.4%)	9 (64.3%)	2 (14.3%)	-
Anemia	3 (21.4%)	9 (64.3%)	2 (14.3%)	-
**Unclassified**				
Highest toxicity	7 (70%)	2 (20%)	1 (10%)	-
CYTOLYSE	2 (20%)	-	-	-
ERUPTION CUTANEE	1 (10%)	-	-	-

Type of toxicity	Grade 1	Grade 2	Grade 3	Grade 4
	N (%)	N (%)	N (%)	N (%)
ERYHTHEME PRURIGINEUX LOMBAIRE	1 (10%)	-	-	-
ILEITE	-	1 (10%)	-	-
LEUCORRHEES	1 (10%)	-	-	-
METRORRAGIES	1 (10%)	1 (10%)	-	-
NEUROPATHIE	1 (10%)	-	-	-
NEUROPATHIE PERIPHERIQUE	1 (10%)	-	-	-
REACTION A LA PERFUSION	1 (10%)	-	-	-
RECTITE	2 (20%)	-	-	-
SARCOIDOSE	1 (10%)	-	-	-
SARCOIDOSE LIKE	1 (10%)	-	-	-
SECHRESSE MUQUEUSE NASALE	1 (10%)	-	-	-
SYNDROME RECTAL	1 (10%)	-	-	-
TROUBLE DE L'ODORAT	1 (10%)	-	-	-
URETERAL INJURIES WITH RENAL FAILURE	-	-	1 (10%)	-
**Metabolism and nutrition disorders**				
Highest toxicity	7 (53.8%)	5 (38.5%)	1 (7.7%)	-
Anorexia	6 (46.2%)	6 (46.2%)	-	-
Hypokalemia	1 (7.7%)	-	1 (7.7%)	-
Hypomagnesemia	-	-	1 (7.7%)	-
Hyponatremia	1 (7.7%)	-	-	-
**Infections and infestations**				
Highest toxicity	8 (100%)	-	-	-
Urinary tract infection	8 (100%)	-	-	-
Vulval infection	1 (12.5%)	-	-	-
**Musculoskeletal and connective tissue disorders**				
Highest toxicity	4 (100%)	-	-	-
Arthralgia	3 (75%)	-	-	-
Chest wall pain	1 (25%)	-	-	-
**Nervous system disorders**				
Highest toxicity	4 (100%)	-	-	-
Dysgeusia	4 (100%)	-	-	-
Headache	2 (50%)	-	-	-
**Reproductive system and breast disorders**				
Highest toxicity	2 (66.7%)	1 (33.3%)	-	-
Pelvic pain	2 (66.7%)	1 (33.3%)	-	-
Uterine pain	1 (33.3%)	-	-	-
**Renal and urinary disorders**				
Highest toxicity	3 (60%)	1 (20%)	1 (20%)	-

Type of toxicity	Grade 1	Grade 2	Grade 3	Grade 4
	N (%)	N (%)	N (%)	N (%)
Acute kidney injury	1 (20%)	-	1 (20%)	-
Cystitis noninfective	1 (20%)	2 (40%)	-	-
Urinary frequency	1 (20%)	-	-	-
Urinary incontinence	1 (20%)	-	-	-
Urinary tract pain	1 (20%)	-	-	-
**Respiratory, thoracic and mediastinal disorders**				
Highest toxicity	4 (80%)	1 (20%)	-	-
Dyspnea	3 (60%)	1 (20%)	-	-
Epistaxis	1 (20%)	-	-	-
Hoarseness	1 (20%)	-	-	-
**Endocrine disorders**				
Highest toxicity	2 (50%)	2 (50%)	-	-
Hyperthyroidism	2 (50%)	1 (25%)	-	-
Hypothyroidism	-	1 (25%)	-	-
**Skin and subcutaneous tissue disorders**				
Highest toxicity	4 (66.7%)	2 (33.3%)	-	-
Dry skin	1 (16.7%)	-	-	-
Pruritus	1 (16.7%)	2 (33.3%)	-	-
Rash maculo-papular	2 (33.3%)	1 (16.7%)	-	-
**Vascular disorders**				
Highest toxicity	2 (50%)	-	2 (50%)	-
Flushing	2 (50%)	-	-	-
Hypotension	-	-	2 (50%)	-
**Ear and labyrinth disorders**				
Highest toxicity	3 (75%)	1 (25%)	-	-
Hearing impaired	1 (25%)	-	-	-
Tinnitus	2 (50%)	1 (25%)	-	-
**Eye disorders**				
Highest toxicity	1 (100%)	-	-	-
Dry eye	1 (100%)	-	-	-

Supplementary Table 2 - Multiplex Immunohistochemistry antibody details

Panel 1						
**Marker**	**Clone**	**Fluorochrome**	**Cat #**	**Company**	**Dilution**	**Validation**
CD3	polyclonal	Opal 650	A0452	Agilent	400	Tonsil
Granzyme B	GrB-7	Opal 540	M7235	Agilent	100	Tonsil
FOXP3	236A/E7	Opal 620	ab20034	Abcam	200	Tonsil
CD11c	2F1C10	Opal 520	60258-1-Ig	Protein tech	10 000	Tonsil
PD-L1	ZR3	Opal 570	Z2002RL	Diagomics	600	Tonsil /Placenta
Cytokeratin	AE1/AE3	Opal 690	M3515	Agilent	200	Breast

Panel 2						
**Marker**	**Clone**	**Fluorochrome**	**Cat #**	**Company**	**Dilution**	**Validation**
Granzyme B	GrB-7	Opal 540	M7235	Agilent	100	Tonsil
FOXP3	236A/E7	Opal 620	ab20034	Abcam	200	Tonsil
CD11c	2F1C10	Opal 650	60258-1-Ig	Protein tech	10 000	Tonsil
KI67	MIB-1	Opal 520	Dako M7240	Agilent	1000	Tonsil
Cytokeratin	AE1/AE3	Opal 690	M3515	Agilent	200	Breast

Panel 3						
**Marker**	**Clone**	**Fluorochrome**	**Cat #**	**Company**	**Dilution**	**Validation**
CD8	C8/144B	Opal 520	M7103	Agilent	100	Tonsil
CD28	EPR22076	Opal 650	ab243228	Abcam	500	Tonsil
CD86	EP1158-37	Opal 570	ab269587	Abcam	50	Tonsil
CD11c	2F1C10	Opal 540	60258-1-Ig	Protein tech	10 000	Tonsil
PD-1	EPR4877(2)	Opal 620	ab137132	Abcam	500	Tonsil
Cytokeratin	AE1/AE3	Opal 690	M3515	Agilent	200	Breast

Supplementary Table 3 - Flow cytometry antibody details

Marker	Clone	Fluorochrome	Cat #	Company	Dilution	Validation/Gated on
CD1c	L161	BV421	331526	Biolegend	1:60	PBMCs
CD3	UCHT1	APC-Cy7	300426	Biolegend	1:50	PBMCs/Lymphocytes
CD3	UCHT1	AF700	300424	Biolegend	1:50	PBMCs/Lymphocytes
CD4	OKT4	BV650	317436	Biolegend	1:100	PBMCs/Lymphocytes
CD8	3B5	PE-Texas Red	MHCD0817	ThermoFisher	1:80	PBMCs/Lymphocytes
CD11c	B-ly6	BUV395	563787	BD	1:50	PBMCs
CD14	M5E2	AF488	557700	BD	1:50	PBMCs/Monocytes
CD16	3G8	BUV737	564434	BD	1:100	PBMCs
CD19	HIB19	AF700	302226	Biolegend	1:50	PBMCs/Lymphocytes
CD25	BC96	BV605	302623	Biolegend	1:40	3-days PHA-stimulated PBMCs/Lymphocytes
CD27	M-T271	PerCP-Cy5.5	356408	Biolegend	1:100	PBMCs
CD45	2D1	APC-Cy7	557833	BD	1:50	PBMCs/Lymphocytes
CD45RA	HI100	BV786	563870	BD	1:120	PBMCs/Lymphocytes
CD56	B159	AF700	557919	BD	1:100	PBMCs/Lymphocytes
CD56	NCAM16.2	BUV395	563554	BD	1:100	PBMCs/Lymphocytes
CD123	6H6	AF647	306024	Biolegend	1:100	PBMCs
CD127	A01905	AF488	351314	Biolegend	1:80	PBMCs/Lymphocytes (CD3+)
CD141	M80	BV786	344116	Biolegend	1:50	overnight LPS-stimulated PBMCs/Monocytes
CD141	M80	PerCP-Cy5.5	344112	Biolegend	1:25	overnight LPS-stimulated PBMCs/Monocytes
CD163	RM3/1	AF647	326508	Biolegend	1:80	overnight IL-10-stimulated PBMCs/Monocytes
CD206	19.2	PE-CF594	564063	BD	1:50	3-days GM-CSF-stimulated PBMCs/Monocytes
EpCAM	9C4	BV785	324238	Biolegend	1:100	HT29 cell line
HLA-DR	L243	BV650	307650	Biolegend	1:120	PBMCs/Monocytes
ICOS	DX29	BUV737	564778	BD	1:25	3-days PHA-stimulated PBMCs/Lymphocytes
ICOS-L	2D3	PE-Cy7	309410	Biolegend	1:25	Burkitt's lymphoma cell line Daud
OX40	Ber-ACT35	PE	350004	Biolegend	1:25	3-days PHA-stimulated PBMCs/Lymphocytes
Ki67	Ki67	BV421	350506	Biolegend	1:50	3-days PHA-stimulated PBMCs/Lymphocytes
PD-L1	MIH1	BV421	563738	BD	1:30	3-days PHA-stimulated PBMCs/Lymphocytes
PD-L1	MIH1	PerCP-eFluor710	46-5983-42	eBioscience	1:50	3-days PHA-stimulated PBMCs/Lymphocytes
PD-1	EH12.2H7	AF647	329910	Biolegend	1:50	PBMCs/Lymphocytes (CD3+)
IgG4 (secondary ab)	HP-6025	Biotin	B3648	Sigma	1:100	
Streptavidin	-	AF647	405237	Biolegend	1:200	

Isotype Controls

Mouse IgG1, κ (OX40 Isotype)	MPC-11	PE	400114	Biolegend	Lot-dependent	-
Mouse IgG1, κ (ICOS Isotype)	X40	BUV737	564299	BD	Lot-dependent	-
Mouse IgG2b, κ (ICOS-L Isotype)	MOPC-11	PE-Cy7	400326	Biolegend	Lot-dependent	-
Mouse IgG1, κ (PD-L1 Isotype)	P3.6.2.8.1	PerCP-eFluor710	25-4714-42	eBioscience	Lot-dependent	-
Mouse IgG1, κ (PD-L1 Isotype)	MOPC-21	BV421	400157	Biolegend	Lot-dependent	-

Other Reagents:

LIVE/DEAD™ Fixable Aqua Dead Cell Stain Kit			L34957	ThermoFisher	1:1000 in PBS	-
Fc block			14-9161-73	eBioscience	1:25 in PBS	-
Foxp3 / Transcription Factor Fixation/Permeabilization Concentrate and Diluent Kit			00-5521-00	eBioscience	Manufacturer's instructions	-

Ensemble, prenons le cancer de vitesse.

EUDRACT: 2016-004689-24
BMS ref. CA209-767
IC 2016-08

NiCOL

A phase-I study of nivolumab in association with radiotherapy and cisplatin in locally advanced cervical cancers followed by adjuvant nivolumab for up to 6 months

Version n° 1.3 –11/09/2017 – (approved by IRB and ANSM)
Version n° 2.1 –22/08/2019 – (approved by IRB and ANSM)

**INVESTIGATOR COORDINATOR**	**Dr Emanuela Romano**<br>Institut Curie<br>26, rue d'Ulm<br>75248 Paris Cedex 05

**SPONSOR**	**Institut Curie**<br>26, rue d'Ulm – 75248 Paris Cedex 05

Ensemble, prenons le cancer de vitesse.

APPROVAL AND RESPONSABILITIES

Title: A phase-I study of nivolumab in association with radiotherapy and cisplatin in locally advanced cervical cancers followed by adjuvant nivolumab for up to 6 months

**Competent Authority**	**Agence Nationale de Sécurité du Médicament et des Produits de Santé (ANSM)**	**Date of authorization: 16/06/2017**
		**Ref. ANSM: 170078A-12**
**Ethics Committee**	**Name of the Committee for the Protection of Persons (CPP):**<br><br>**CPP SUD-EST VI Clermont-Ferrand**	**Date of approval: 21/09/2017**
		**Ref. CPP: AU 1316**

**Steering Committee**	Pr Durdux and Drs Minsat, Rodrigues, and Romano
**Clinical Trial Manager**	Souhir Neffati
**Translational Research Coordinator**	Maud Kamal, PhD

**Writing Committee Coordinator**	Dr Manuel Rodrigues
**Writing Committee**	Drs Bazire, Durdux, Kamal, Minsat, Rodrigues, Romano, and Savignoni, S Armanet, A Rohel, S Neffati

**Names and Responsibilities**	**Contact**	**Date** ***(dd-mm-yyyy)***	**Signature**
**General Director of the Hospital Group**	Pr. Pierre Fumoleau		
**Coordinator**	Dr. Emanuela Romano		
**Biostatistician**	Dr. Alexia Savignoni		

institutCurie
Ensemble, prenons le cancer de vitesse.

SYNOPSIS – PROTOCOL N° IC 2016-08
EUDRACT N°: 2016-004689-24

**A) Clinical Study Identification**	
**Protocol Number**: IC 2016-08<br><br>**Version and Date**: Version 2.1 ; 22/08/2019	
**Study Title**: A phase-I study of nivolumab in association with radiotherapy and cisplatin in locally advanced cervical cancers followed by adjuvant nivolumab for up to 6 months	
**Short Title**: NiCOL	
**Coordinator**: Dr Emanuela Romano	
**Estimated Number of Centers**: 3	**Number of Patients**: 21 (maximum)

**B) Sponsor Identification**
Institut Curie<br>26, rue d'Ulm - 75248 Paris Cedex 05
Pierre FUMOLEAU, MD PhD<br>General Director of the Hospital Group<br>Institut Curie<br>26, rue d'Ulm - 75248 Paris Cedex 05<br>**Phone:** +33-1-5624-5765 – **Fax:** +33-1-5310-4029 – **E-mail:** drci.promotion@curie.fr

C) General Study Information

Indication: Patients with confirmed locally advanced cervical cancer, i.e. FIGO stages IB2 to IVA squamous-cell carcinoma or adenocarcinoma, with indication for radiotherapy and cisplatin-based chemotherapy with a curative intent as confirmed by a multidisciplinary board.

Methodology: This is a multicenter, non-randomized, open-label, dose-confirmatory, phase-I cohort study.

Primary objective: Confirm the recommended phase-II dose of nivolumab in association with pelvic +/- para-aortic chemoradiation therapy in patients with locally advanced cervical cancer.

Secondary objectives:

- Assess the objective response rate (ORR) to the association of nivolumab and chemoradiation therapy;
- Assess the 2-year progression free survival (PFS) rate;
- Assess the 2-year disease free survival (DFS) rate;
- Assess the overall safety profile of the association of nivolumab and chemoradiation therapy;
- Identify potential biomarkers associated with safety and objective response.

Inclusion Criteria:

1. Adult patients at least 18 years of age;
2. Ability to understand and the willingness to sign a written informed consent document.;
3. Eastern Cooperative Oncology Group (ECOG) Performance Status 0 or 1;
4. Histologically confirmed locally advanced cervical cancer, i.e. FIGO stages IB2 to IVA, squamous-cell carcinoma or adenocarcinoma, with indication for radiotherapy and cisplatin-based chemotherapy with a curative intent as confirmed by a multidisciplinary board including a radiation oncologist. PD-L1 expression on tumor will not be required for inclusion; (staging may include [18F]-fluorodeoxyglucose (FDG) PET-CT and/or para-aortic dissection in accordance with usual practice in each investigational center and at the Investigator's discretion);
5. Disease amenable to biopsy since three tumor samples are mandatory prior to treatment;
6. Laboratory values at Screening must meet the following criteria :
   - ✓ neutrophils ≥ 1.0 x 10^9/L,
   - ✓ lymphocytes ≥ 0.5 x 10^9/L,
   - ✓ platelets ≥ 100 x 10^9/L,
   - ✓ hemoglobin ≥ 8.0 g/dL,
   - ✓ creatinine ≤ 2 times the upper limit of normal (ULN),
   - ✓ aspartate aminotransferase (AST) ≤ 3 ULN,
   - ✓ alanine aminotransferase (ALT) ≤ 3 x ULN,
   - ✓ total bilirubin ≤ 1.5 x ULN (≤ 3 x ULN if genetically documented Gilbert's syndrome).
7. For women with child-bearing potential, negative blood or urinary pregnancy test within 24 hours of initiation of nivolumab, as well as appropriate method of contraception throughout the study ;
8. Affiliated to the French Social Security System.

Non-inclusion Criteria:

1. Metastases (except pelvic and/or para-aortic nodal metastases) ;
2. Peritoneal carcinosis;
3. Sensory or motor neuropathy ≥ grade 2;
4. Active or recent history of known autoimmune disease or recent history of a syndrome that required systemic corticosteroids or immunosuppressive drugs, except for :
   - hydrocortisone, which is permitted at physiological doses;
   - syndromes that would not be expected to recur in the absence of an external trigger, e.g. glomerulonephritis;
   - vitiligo or autoimmune thyroiditis;
5. Type-1 or type-2 diabetes;
6. History of or current immunodeficiency disease, including known history of infection with human immunodeficiency virus;
7. Prior systemic treatment or radiotherapy for cervical cancer;

8. Prior allogeneic stem cell transplantation;
9. Prior immunotherapy, including tumor vaccine, cytokine, anti-CTLA4, anti-PD-1, anti-PD-L1 or similar agents;
10. Any non-oncologic vaccine for prevention of infectious disease within 28 days prior to inclusion, including but not limited to measles, mumps, rubella, chicken pox, yellow fever, seasonal influenza, H1N1, rabies, BCG, and typhoid vaccine;
11. Positive serology for hepatitis B surface antigen;
12. Positive for hepatitis-C ribonucleic acid on polymerase chain reaction;
13. Active infection requiring therapy;
14. History of idiopathic pulmonary fibrosis (including pneumonitis), drug-induced pneumonitis, organizing pneumonia or evidence of active pneumonitis on chest CT-scan at Screening;
15. History of malignancy (excepting non-melanoma skin cancer) unless complete remission was achieved at least 3 years prior to inclusion and no additional therapy is required or planned during the study;
16. Underlying medical condition that, in the Investigator's opinion, could render the administration of the study treatment hazardous; additional severe and/or uncontrolled concurrent disease;
17. Concomitant use of other investigational drugs;
18. Pregnancy or breastfeeding.

Primary Endpoint:

The primary endpoint is the rate of occurrence of dose-limiting toxicity (DLT) within 11 weeks after the initiation of treatment.

DLT is defined as any of the following treatment-related adverse events or laboratory abnormalities, graded according to National Cancer Institute Common Terminology Criteria for Adverse Events (NCI-CTCAE) version 4.03:

- non-hematological toxicity ≥ grade 3;
- immune-related adverse event ≥ grade 3;
- symptomatic immune-related adverse event ≥ grade 2 resistant to optimal supportive care for > 7 days;
- dosing delay in RT ≥ 1 week due to toxicity related to nivolumab, chemotherapy or RT;
- colitis or diarrhea ≥ grade 3.

Secondary Endpoints:

The secondary endpoints for assessment of the efficacy of the association of nivolumab and radio-chemotherapy are ORR, PFS and DFS.

ORR is defined as the proportion of all subjects whose best response is either a complete response or a partial response. ORR will be assessed using the Response Evaluation Criteria In Solid Tumors (RECIST), version 1.1, assessed after the end of RT and before brachytherapy and again up to 2 months after brachytherapy.

PFS is defined as the length of time from the start of treatment to disease progression or death,

regardless of the cause of death.

DFS is defined as the length of time from the start of complete response to the time of relapse from complete response. DFS applies only to patients in complete response.

The secondary endpoints for assessment of the overall safety profile of the association of nivolumab and radio-chemotherapy are adverse events (AEs) and serious adverse events (SAEs) from the first intake of the IMP until 100 days after the last intake of the IMP, with a particular focus on local versus systemic immune-related AEs.

The secondary endpoints for the identification of potential biomarkers associated with clinical response and/or toxicities are:

- tumor PD-L1 immunohistochemistry;
- immunohistochemistry to evaluate presence and phenotype of tumor-infiltrating lymphocytes and tumor-associated macrophages;
- HPV typing and identification of the viral integration loci in order to make possible non-invasive tumor monitoring with circulating tumor DNA and HPV DNA;
- immunomonitoring of circulating blood lymphocytes with extensive immune panels;
- gene status of known oncogenes and tumor-suppressor genes with next-generation DNA sequencing associated with copy number and heterozygosity assessment;
- vaginal microbiota sampling for exploratory study.

D) Description of Investigational Medicinal Product

Drug

International Nonproprietary Name	Trade Name	Pharmaceutical Form	Route of Administration	Dosage
Nivolumab	Opdivo®	Solution for infusion	Intravenous	240 mg flat dose q2wk
Cisplatin	-	Solution for infusion	Intravenous	40 mg/m^2

Ensemble, prenons
le cancer de vitesse.

Therapeutic scheme:

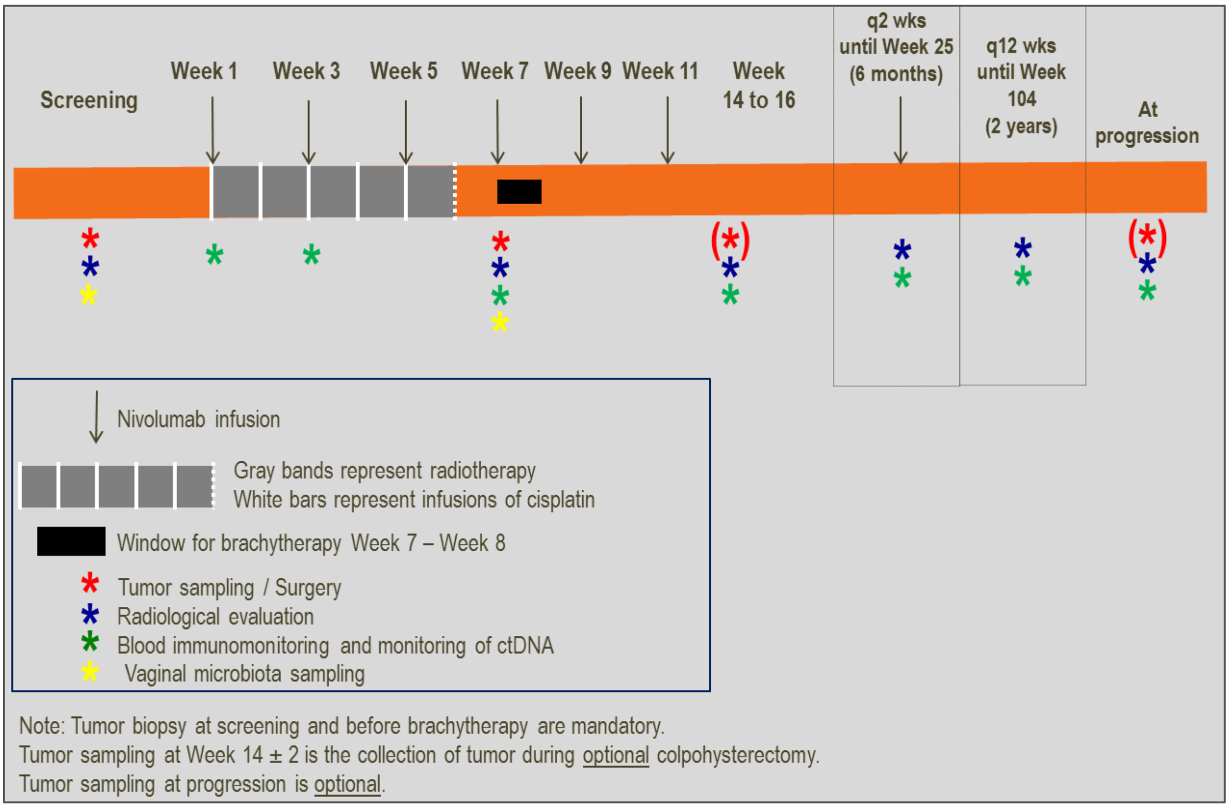

Treatment duration: 6 months

E) Statistical Analysis

Sample size:

The minimum sample size will be 15 evaluable patients and the maximum will be 21 evaluable patients.
Patients who failed to complete the first 11 weeks of treatment (= DLT evaluation period) for a reason other than DLT, or who received a reduced dose of study drugs (<70% of the planned dose of nivolumab/chemotherapy/radiotherapy) for a reason other than DLT, will be considered as not evaluable for the primary toxicity endpoint assessment.
Non evaluable patient should be replaced.
The study will use a 3+3 design to confirm the dose of nivolumab, i.e. 240 mg flat dose q2wk.
Thus, the DLT assessment phase will include up to 6 patients at the given dose.
An expansion cohort of 9 patients will then open to collect additional data on the safety profile of nivolumab in association with radio-chemotherapy and as adjuvant therapy in the treatment of locally advanced cervical cancer.

Statistical analysis:

Safety analysis (primary analysis)

The Phase I main analysis set will include only patients deemed evaluable for the DLT evaluation (DLT population).
Safety analyses will include:

- Maximum CTCAE grade (Version **CTCAE v4.03**) for adverse events,
- Serious adverse events during the treatment period and during DLT period,
- Study discontinuation due to adverse event,
- Death occurring during the study treatment period and during DLT period.

Secondary efficacy and safety objectives analyses

The objective response rate, progression-free survival rate, and disease-free survival rate will be the secondary endpoints. The analyses will be exploratory and descriptive, and will be performed on all patients who received at least one administration of the recommended dose of nivolumab, whether or not they are evaluable for DLT, in order not to overestimate the response rate or long-term criteria as progression-free survival, by excluding patients who progress or die during the DLT window.

The ORR will be given with 95% exact confidence interval with binomial distribution. PFS and DFS will be estimated by the Kaplan-Meier method and given with their 95% confidence interval.

F) Study Duration
**Inclusion Period:** 30 months
**Treatment and Follow-up Period:** 24 months
**Estimated Total Study Duration:** 54 months

Ensemble, prenons le cancer de vitesse.

TABLE OF CONTENTS

1. Introduction and Rationale 13

1.1 Introduction 13
1.2 Rationale of the Study 14

2. Study Objectives 15

2.1 Primary Objective 15
2.2 Secondary Objectives 15

3. Study Design 15

3.1 Data Safety Monitoring Board (DSMB) 16
3.2 Independent Data Monitoring Committee (IDMC) 16
3.3 Primary Endpoint 17
3.4 Secondary Endpoints 17

4. Patient Selection and Registration 18

4.1 Selection Criteria 18
4.1.1 Inclusion Criteria 18
4.1.2 Non-inclusion Criteria 18
4.2 Patient Registration 19
4.3 Patient Withdrawal and Study Termination 19
4.3.1 Patient Withdrawal 20
4.3.2 Patient's end of participation 20
4.3.3 Study Termination 21

5. Study Treatments 21

5.1 Administration of Study Treatment 21
5.1.1 External Radiotherapy 21
5.1.2 Brachytherapy 23
5.1.3 Chemotherapy 23
5.1.4 Additional Local Treatment 23
5.1.5 Handling of Toxicities related to Study Treatments 23
5.1.6 Record of Administration 24
5.1.7 Prohibited Concomitant Medication 24
5.2 Investigational Medicinal Product 24
5.2.1 Description of the IMP 24
5.2.2 Dosage Form and Packaging 24
5.2.3 Labeling 24
5.2.4 Storage 24
5.2.5 Dose Calculation and Administration 25
5.2.6 Handling of Toxicities related to IMP 25
5.2.7 IMP Accountability 25
5.2.8 IMP Handling and Dispensing 25
5.2.9 IMP Destruction 26

6. Schedule of Study Assessments and Visits 26

6.1 Procedures to be Performed during Screening Period 27
6.2 Procedures to be Performed during Treatment Period 28
6.3 Procedures to be Performed during Follow-up Period 29
6.4 Procedures to be Performed at End-of-study Visit 30

7. Efficacy Assessments 30

7.1 Response to Treatment 30

8. Molecular, Tumor Microenvironment and Immune Analyses 31

Ensemble, prenons
le cancer de vitesse.

8.1 Molecular Analyses on Tumor Samples 31
8.2 Molecular Analyses on ctDNA 31
8.3 Analyses on Tumor Microenvironment 31
8.4 Immune Analyses 31

9. Safety Assessments 31

9.1 Safety 31
9.2 Definition of Adverse Event 32
9.3 Definition of Serious Adverse Event 32
9.4 Definition of Adverse Reaction 33
9.5 Definition of Suspected Unexpected Serious Adverse Reaction 33
9.6 Severity Criteria 33
9.7 Methods and Timeframes for Collecting Safety Data 34
9.8 Reporting SAEs 34
9.9 Serious Adverse Event Follow-up 35

10. Statistical Considerations 35

10.1 Sample Size and Power Considerations 35
10.2 Statistical Analysis 36
10.2.1 Primary Safety Analysis 36
10.2.2 Analyses of Secondary Efficacy and Safety Endpoints 36

11. Data Protection and Handling of Confidentiality 37

12. Quality Assurance and Quality Control 37

13. Ethical and Regulatory Considerations 37

13.1 Ethics Committee (CPP) 38
13.2 Competent Authority 38
13.3 Information and Consent of Participants 38
13.4 Responsibilities of the Sponsor 39
13.5 Responsibilities of the Investigator 39
13.6 Federation of Patient Committees for Clinical Research in Oncology 40

14. Data Review and Data Management 40

15. Financial Information and Study Insurance 40

16. Publication of Results 41

17. References 42

18. Appendices 43

Appendix 1 – Flow Chart 44
Appendix 2 – ECOG Performance Status Scale 45
Appendix 3 – Toxicity Criteria (NCI CTC-AE) 46
Appendix 4 - Management of Toxicities related to Study Treatments 47
Appendix 5 - Procedures for Tumor Sampling 50

Glossary of Abbreviations

AE	adverse event
ALT	alanine aminotransferase
ANSM	French National Agency for the Safety of Medicines and Healthcare Products
AST	aspartate aminotransferase
AT	aminotransferase
BECT	Unicancer Office of Clinical and Therapeutic Studies
CGH	comparative genomic hybridization
CPP	Committee for the Protection of Persons (Ethics Committee)
CT	computed tomography
CTC-AE	Common Toxicity Criteria - Adverse Events
CRA	Clinical research associate
CTV	clinical target volume
DLT	dose-limiting toxicity
DNA	deoxyribonucleic acid
DILI	Drug Induced Liver Injury
DSMB	Data Safety Monitoring Board
ECOG	Eastern Cooperative Oncology Group
eCRF	electronic case report form
EDTA	ethylene diamine tetraacetic acid
EMEA	European Medicines Agency
EQD2	Equivalent Dose in 2 Gy Fraction
FCPRCC	Federation of Patient Committees for Clinical Research in Oncology
FDG	Fluorodeoxyglucose
FFPE	formalin-fixed paraffin-embedded
FIGO	International Federation of Gynecology and Obstetrics
HDR	high dose rate
GTV	gross tumor volume
Gy	Gray
HPV	human papillomavirus
HR	hazard ratio
IB	investigator brochure
IDMC	Independent Data Monitoring Committee
IEC	Independent Ethics Committee (see also CPP)
IMP	investigational medicinal product
ITV	internal target volume
IV	intravenous
LNCC	National Anticancer League

Ensemble, prenons le cancer de vitesse.

LOH	loss of heterozygosity
MRI	magnetic resonance imaging
NCI	National Cancer Institute
PD-1	programmed cell-death protein 1
PD-L1	programmed cell-death ligand 1
PDR	Pulsed dose rate
PET	positron emission tomography
PPK	population pharmacokinetics
PTV	Planned Target Volume
q2wk	every two weeks
RECIST	Response Evaluation Criteria In Solid Tumors
RT	radiation therapy
SAE	serious adverse event
SAR	serious adverse reaction
SCC	Squamous Cell Carcinoma
SNP	single nucleotide polymorphism
SPC	Summaries of Product Characteristics
SUSAR	suspected unexpected serious adverse reaction
ULN	upper limit of normal
USP	United States Pharmacopeia (standard reference)

1. Introduction and Rationale

1.1 Introduction

Cervical cancer has an incidence of around 3400 new cases/year in France and is responsible for about 1000 deaths/year [1]. Cervical cancer is staged clinically using the criteria developed by the International Federation of Gynecology and Obstetrics (FIGO), which serve as a basis for determining treatment modalities. Locally advanced cervical cancer, i.e. FIGO stages IB2 to IVA, has a poor prognosis and historically has been treated with pelvic radiotherapy (RT). Moreover, the relapse rate of cervical cancer ranges between 11% and 22% in FIGO stages IB–IIA and between 28% and 64% in FIGO stages IIB–IVA. Recurrence of cervical cancer is difficult to treat because of induced chemo-resistance and toxicities of local therapies. Effective adjuvant treatments are therefore desperately needed, however, since pelvic RT dramatically reduces bone marrow function, further addition of cytotoxic agents may not be optimal. Novel immunotherapeutic strategies are needed to improve the prognosis of cervical cancer patients.

The principal etiological factor in cervical cancer is persistent infection with human papillomavirus (HPV), a DNA virus that can infect the genitals, anus, mouth or throat. In general, HPV-induced cancers are rational targets for immune therapy because they express specific oncoproteins, which are nonself antigens and therefore good immunologic targets. Cervical cancers express up to nine HPV-encoded proteins, however, the E6 and E7 viral proteins are of particular importance because they induce malignant transformation of cells and are required for survival of the transformed cells. Better clinical outcomes in HPV-associated cancers have been shown to correlate with T-cell reactivity against E6 and E7, again suggesting their potential as therapeutic targets [2,3]. In addition, they are capable of inactivating p53 and RB, leading to genomic instability and the appearance of neo-antigens. Recently, an immune modulator, imiquimod, has shown clear evidence of activity in HPV-induced tumors. The use of topical imiquimod in high-grade vulvar intraepithelial neoplasia has been reported with a complete response in 34% of patients over a 12-month period [4].

Another immune modulator, nivolumab, is a fully human monoclonal antibody that targets the programmed cell-death protein 1 (PD-1) cell surface-membrane receptor. PD-1 is a negative regulatory molecule expressed by activated T and B lymphocytes. Binding of PD-1 to its ligands, programmed cell-death ligands 1 (PD-L1) and 2 (PD-L2), results in the down-regulation of lymphocyte activation. Thus, inhibition of the interaction between PD-1 and its ligands promotes immune responses and antigen-specific T-cell responses to foreign antigens as well as self-antigens.

Nivolumab as monotherapy is registered in a number of countries, including the United States, and in Europe, for the treatment of unresectable or metastatic melanoma, previously treated metastatic non-small cell lung cancer and previously treated advanced clear-renal cell carcinoma, as well as for the treatment of classical Hodgkin's lymphoma in the United States. The recommended dose of nivolumab as monotherapy in these indications is 240 mg flat dose q2w administered intravenously over 30 minutes every 2 weeks (q2wk).

Various studies have shown that, in 20 to 30% of cervical tumors, PD-L1 expression is present suggesting that pharmacological blockade of the inhibitory PD-1/PD-L1 pathway with nivolumab may be worth considering in the treatment of these tumors [5,6]. Anti-PD-1 and anti-PD-L1 agents have shown activity in a wide range of tumors with an overall response rate of about 20 to 25% with higher

response rates when PD-L1 was overexpressed. Previous data demonstrated an endogenous antitumor response in patients with cervical cancer, which can be boosted in part by the antigenic release triggered by radio-chemotherapy [2,3]. RT can also up-regulate the expression of PD-L1 on cancer cells.

Indeed, RT induces tumor cell death, and also induces tumor-specific adaptive immune responses. Recent reports have shown synergies with abscopal responses to RT, i.e. responses distant from the site of RT, associated with immune checkpoint blockers [7-10]. One reason for relapse is that the antitumor immunity induced by RT is not maintained. In a recent study, Deng et al. investigated PD-L1 expression in breast and colorectal xenografts [11]. PD-L1 was up-regulated in xenografts after RT suggesting that changes in the tumor immune microenvironment after RT may limit infiltration of effector T-cells. In that study, RT alone and anti-PD-L1 alone had minimal effects on tumor growth while association of the two led to tumor regression and prevented recurrence. Mice treated with the association were also resistant to tumor rechallenge, indicating that the association results in extended anti-tumor immunity. Thus, concomitant treatment with nivolumab is likely to enhance the antitumor response and results in long-lasting clinical benefit. Of note, synergy between RT and PD-L1 blockade was dependent on the modulation of myeloid-derived suppressor cells in the tumor.

Immune checkpoint blockade, using antibodies that target cytotoxic T-lymphocyte-associated antigen 4 (CTLA-4) or the PD-1/PD-L1 pathway, arguably represents one of the most important recent advances in oncology. A major challenge in addressing the mechanisms of response to checkpoint inhibition and their combination with other therapeutic modalities relates to the extraordinary complexity of immunoregulatory systems in the tumor microenvironment , where tumor cells and an ever-growing list of tumor-infiltrating host leukocytes and stromal cells (including blood and lymphatic endothelial cells, and fibroblasts) can suppress or even block T-cell homing, engraftment or effector function, using diverse but often overlapping mechanisms (recently reviewed). Among immunosuppressive leukocytes, regulatory T-cells, regulatory B-cells, NK cells and γδ T-cells, myeloid-derived suppressor cells, plasmacytoid dendritic cells, tumor-associated macrophages and Tie2-expressing monocytes can suppress or tolerize T-cells in tumors. Further complicating the landscape, the tumor vasculature, stromal lymphatic vessels, and the sentinel lymph node may all play critical immunoregulatory roles in tumors. In-depth analysis of the tumor microenvironment and the immunological response are therefore needed in order to gain a better understanding of the mechanisms associated with response, as well as with toxicities.

1.2 Rationale of the Study

To date, the majority of clinical trials on checkpoint inhibitors have tested these agents as monotherapy, and the next logical step is to evaluate rational therapeutic associations. The aim of the NiCOL study is to assess the safety of nivolumab in association with chemoradiation therapy and to gain initial insight into its efficacy in association with the current standard of care, including chemoradiation.

Pelvic RT with concurrent cisplatin-based chemotherapy has been the standard of care for the past 15 years. On the basis of 13 studies that compared chemoradiation therapy to the same radiotherapy alone, there was a 6% improvement in 5-year survival (from 60% to 66%) with chemoradiation therapy (hazard ratio (HR) = 0.81, $p < 0.001$) and an 8% improvement in 5-year disease-free survival, compared to RT alone. However, the improvement in absolute 5-year survival was only 3% for patients

with stage IIIB–IVa disease. Moreover, the relapse rate of cervical cancer ranges between 11% and 22% in FIGO stages IB–IIA and between 28% and 64% in FIGO stages IIB–IVA [12].

2. Study Objectives

2.1 Primary Objective

The primary objective is to confirm the recommended phase-II dose of nivolumab in association with pelvic +/- para-aortic chemoradiation therapy in patients with locally advanced cervical cancer.

2.2 Secondary Objectives

The secondary objectives are:

- to assess the objective response rate (ORR) to the association of nivolumab and chemoradiation therapy;
- to assess the 2-year progression free survival (PFS) rate;
- to assess the 2-year disease free survival (DFS) rate;
- to assess the overall safety profile of the association of nivolumab and chemoradiation therapy;
- to identify potential biomarkers associated with safety and objective response.

3. Study Design

This is a multicenter, non-randomized, open-label, dose-confirmatory, phase-I cohort study on the safety of nivolumab in association with pelvic +/- para-aortic chemoradiation therapy and as adjuvant therapy in the treatment of locally advanced cervical cancer.

The study will use a 3+3 design to confirm the dose of nivolumab, i.e. 240 mg flat dose q2wk in association with chemoradiation therapy from Week 1 to Week 5 and nivolumab alone (+/- brachytherapy) from Week 7 to Week 11. The first cohort of 3 patients will receive nivolumab at 240 mg flat dose q2wk in combination with chemoradiation therapy from Week 1 to Week 5 and nivolumab alone (+/- brachytherapy) from Week 7 to Week 11 and, if DLT is considered acceptable, i.e. present in 1 or 0 patients, an additional cohort of 3 patients will be included until 6 patients have been treated. If DLT is considered unacceptable, i.e. present in 2 or 3 patients, the first cohort will be stopped and a new cohort of 3 patients will be set up. The second cohort will receive nivolumab at 1 mg/kg q2wk in association with chemoradiation therapy from Week 1 to Week 5 and nivolumab alone (+/- brachytherapy) from Week 7 to Week 11 and, if DLT is considered acceptable, i.e. present in 1 or 0 patients, an additional cohort of 3 patients will be included until 6 patients have been treated. If DLT is considered unacceptable, i.e. present in 2 or 3 patients, the second cohort will be stopped and the study will terminate.

Thus, the DLT assessment phase will include up to 6 patients at the given dose. DLT will be considered acceptable if 1 or 0 patients among the first 6 DLT evaluable patients develop DLT. An

expansion cohort of 9 patients will then open to collect additional data on the safety profile of nivolumab in association with chemoradiation therapy and as adjuvant therapy in the treatment of locally advanced cervical cancer.

Per the IB a flat dose of Nivolumab 240 mg q2w was selected since it is identical to a dose of 3 mg/kg for subjects weighing 80 kg, the observed median body weight in nivolumab-treated cancer patients. Using a PPK model, the overall distributions of nivolumab exposures (Cavgss, Cminss, Cmaxss, and Cmin1) are comparable after treatment with either 3 mg/kg or 240 mg nivolumab. The predicted range of nivolumab exposures (median and 90% prediction intervals) resulting from a 240 mg flat dose across the 35 to 160 kg weight range is maintained well below the corresponding exposures observed with the well tolerated 10 mg/kg nivolumab q2w dosage (refer to the Investigational Brochure N°18 dated 25-Jun-2019). The 240 mg flat dose of nivolumab has been chosen in the NCT02764593 and NCT02768558 clinical trials, which combine nivolumab to chemotherapy ± radiation therapy [13].

The expected duration of each patient's participation in the study is expected to be 24 months. The overall duration of recruitment is expected to be 30 months.

3.1 Data Safety Monitoring Board (DSMB)

Decisions with regard to addition of cohorts, to dose reduction, if any, and to study termination will be taken conjointly by a Data Safety Monitoring Board.

This Committee will be composed by the Sponsor (the methodologist, a person in charge of the pharmacovigilance, and the project manager), the principal investigator in each investigational center and/or co-coordinating investigators, and the Steering Committee.

The role of the DSMB will be:

- to review the patient accrual,
- to monitor the toxicity of treatments,
- to comment the reporting of toxicity and adverse events,
- to review the dose escalation results from all the patients to confirm the recommended dose for the phase II part of the study.

The Safety Review Committee determines when an Independent Data Monitoring Committee (IDMC) is needed.

3.2 Independent Data Monitoring Committee (IDMC)

An independent Data Monitoring Committee (IDMC) will be composed of three or five independent experts who have no conflict of interest and agree with the outline of the protocol. None of the members of the IDMC should be among the participants of the trial.

The role of the IDMC will be:
- to review the patient accrual,

- to monitor the toxicity of treatments
- to comment the reporting of toxicity and adverse events
- to treatment response data

All data presented at these meetings will remain confidential.

This IDMC may be requested by the Data Safety Monitoring Board in case of an interim analysis has been performed by the statistician of the trial, for any serious decision or complications following this meeting. The IDMC will report to the study Coordinator and may recommend changes in the conduct of the trial as early stopping, change, continuation or extension of the trial.

3.3 Primary Endpoint

The primary endpoint is the rate of occurrence of dose-limiting toxicity (DLT) within 11 weeks after the initiation of treatment.

DLT is defined as any of the following treatment-related adverse events or laboratory abnormalities, graded according to National Cancer Institute Common Terminology Criteria for Adverse Events (NCI-CTCAE) version 4.03:

- non-hematological toxicity ≥ grade 3;
- immune-related adverse event ≥ grade 3;
- symptomatic immune-related adverse event ≥ grade 2 resistant to optimal supportive care for > 7 days;
- dosing delay in RT ≥ 1 week due to toxicity related to nivolumab, chemotherapy or RT;
- colitis or diarrhea ≥ grade 3.

3.4 Secondary Endpoints

The secondary endpoints for assessment of the efficacy of the association of nivolumab and radio-chemotherapy are ORR, PFS and DFS.

ORR is defined as the proportion of all subjects whose best response is either a complete response or a partial response. ORR will be assessed using the Response Evaluation Criteria In Solid Tumors (RECIST), version 1.1, assessed after the end of RT and before brachytherapy and again up to 2 months after brachytherapy.

PFS is defined as the length of time from the start of treatment to disease progression or death, regardless of the cause of death.

DFS is defined as the length of time from the start of complete response to the time of relapse from complete response. DFS applies only to patients in complete response.

The secondary endpoints for assessment of the overall safety profile of the association of nivolumab and chemoradiation therapy are adverse events (AEs) and serious adverse events (SAEs) from the first intake of the IMP until 100 days after the last intake of the IMP, with a particular focus on local versus systemic immune-related AEs.

The secondary endpoints for the identification of potential biomarkers associated with clinical response and/or toxicities are:

- tumor PD-L1 immunohistochemistry;
- immunohistochemistry to evaluate presence and phenotype of tumor-infiltrating lymphocytes and tumor-associated macrophages;
- HPV typing and identification of the viral integration loci in order to make possible non-invasive tumor monitoring with circulating tumor DNA and HPV DNA;
- immunomonitoring of circulating blood lymphocytes with extensive immune panels;
- gene status of known oncogenes and tumor-suppressor genes with next-generation DNA sequencing associated with copy number and heterozygosity assessment.

4. Patient Selection and Registration

4.1 Selection Criteria

Patients must fulfill all of the inclusion criteria and none of the non-inclusion criteria.

4.1.1 Inclusion Criteria

1. Adult patients at least 18 years of age;
2. Ability to understand and the willingness to sign a written informed consent document;
3. Eastern Cooperative Oncology Group (ECOG) Performance Status 0 or 1 (see Appendix 2; p.45);
4. Histologically confirmed locally advanced cervical cancer, i.e. FIGO stages IB2 to IVA, squamous-cell carcinoma or adenocarcinoma, with indication for radiotherapy and cisplatin-based chemotherapy with a curative intent as confirmed by a multidisciplinary board including a radiation oncologist. PD-L1 expression on tumor will not be required for inclusion;
5. Disease amenable to biopsy since three tumor samples are mandatory prior to treatment;
6. Laboratory values at Screening must meet the following criteria:
   - neutrophils ≥ 1.0 x 10^9/L,
   - lymphocytes ≥ 0.5 x 10^9/L,
   - platelets ≥ 100 x 10^9/L,
   - hemoglobin ≥ 8.0 g/dL,
   - creatinine ≤ 2 times the upper limit of normal (ULN),
   - aspartate aminotransferase (AST) ≤ 3 ULN,
   - alanine aminotransferase (ALT) ≤ 3 x ULN,
   - total bilirubin ≤ 1.5 x ULN (≤ 3 x ULN if genetically documented Gilbert's syndrome).
7. For women in child-bearing potential, negative blood or urinary pregnancy test within 24 hours of initiation of nivolumab, as well as appropriate method of contraception throughout the study;
8. Affiliated to the French Social Security System.

4.1.2 Non-inclusion Criteria

1. Metastases (except pelvic and/or para-aortic nodal metastases);

2. Peritoneal carcinosis;
3. Sensory or motor neuropathy ≥ grade 2;
4. Active or recent history of known autoimmune disease or recent history of a syndrome that required systemic corticosteroids or immunosuppressive drugs, except for:
   - hydrocortisone, which is permitted at physiological doses;
   - syndromes that would not be expected to recur in the absence of an external trigger, e.g. glomerulonephritis;
   - vitiligo or autoimmune thyroiditis;
5. Type-1 or type-2 diabetes;
6. History of or current immunodeficiency disease, including known history of infection with human immunodeficiency virus;
7. Prior systemic treatment or radiotherapy for cervical cancer;
8. Prior allogeneic stem cell transplantation;
9. Prior immunotherapy, including tumor vaccine, cytokine, anti-CTLA4, anti-PD-1, anti-PD-L1 or similar agents;
10. Any non-oncologic vaccine for prevention of infectious disease within 28 days prior to inclusion, including but not limited to measles, mumps, rubella, chicken pox, yellow fever, seasonal influenza, H1N1, rabies, BCG, and typhoid vaccine;
11. Positive serology for hepatitis B surface antigen;
12. Positive for hepatitis-C ribonucleic acid on polymerase chain reaction;
13. Active infection requiring therapy;
14. History of idiopathic pulmonary fibrosis (including pneumonitis), drug-induced pneumonitis, organizing pneumonia or evidence of active pneumonitis on chest CT-scan at Screening;
15. History of malignancy (excepting non-melanoma skin cancer) unless complete remission was achieved at least 3 years prior to inclusion and no additional therapy is required or planned during the study;
16. Underlying medical condition that, in the Investigator's opinion, could render the administration of the study treatment hazardous; additional severe and/or uncontrolled concurrent disease;
17. Concomitant use of other investigational drugs;
18. Pregnancy or breastfeeding.

4.2 Patient Registration

After signature of the informed consent form and validation of the initial Screening assessments and selection criteria, eligible patients will be registered by completing the patient registration form in accordance with the Manual of Study Procedures.

4.3 Patient Withdrawal and Study Termination

Patients will participate to the study for 2 years, i.e. until Week 104. After Week 104, patients will be then considered out of study and will be followed in accordance with usual practices in each center.

4.3.1 Patient Withdrawal

Patients may choose to withdraw from the study, or may be withdrawn from the study at the discretion of the Investigator, at any time. Possible reasons for withdrawal include:

- withdrawal of consent by patient;
- patient lost to follow-up;
- death;

The reason for withdrawal will be recorded in the case report form that need to be signed by the investigator.

If the patient withdraws her consent, she is not required to provide a reason, however, the Investigator must ensure that the withdrawal is not related to an AE. The Investigator must ensure that patients who withdraw or are withdrawn due to AEs receive appropriate care and follow-up.

In so far as possible, the Investigator must perform an end-of-study visit on all patients who withdraw or are withdrawn from the study.

Patients who withdraw or who are withdrawn from the study will not be eligible for re-inclusion.

Patients who withdraw or who are withdrawn from the study will be replaced only if, at the time of withdrawal, they are not evaluable for the DLT assessment (as defined in section 10.1; p. 35)

If a patient withdraws her consent, no further data will be collected after the date of withdrawal. Only data collected prior to withdrawal of consent will be used in the analyses unless the patient voluntarily withdraws consent for use of the data collected.

4.3.2 Patient's end of participation

Possible reasons for patient's end of participation include:

- Dose Limited Toxicities (DLT);
- adverse events (AEs);
- disease progression or relapse
- withdrawal of participation by patient;
- continuation in study treatment not considered to be in patient's best interest.

The reason for end of treatment will be recorded in the case report form.
If the patient withdraws her participation (protocol schedule), she is not required to provide a reason.
When interruption of participation into the study is decided for a patient,

Patients for who interruption of study treatment is decided (without withdrawal of their consent and without progression) are allowed to continue their participation to the study and will enter into follow up phase until two years from start of study treatments.

Patient for who interruption of follow-up is decided without withdrawal of their consent will be then considered out of study and will be followed in accordance with usual practices in each center. Clinical outcome will be followed until two years and the incidence of second cancer will be followed until 5 years after initiation of study treatments.

In case of progression, the patient will be considered out of study and the Investigator must perform an end-of-study visit.

If a patient is considered to be non-evaluable for DLT evaluation of the dose level (from S1 to S11) or If a disease progression appears between S1 to S11, patient should be replaced (as defined in section 10.1; p. 35).

4.3.3 Study Termination

The Sponsor may terminate the study at any time for reasonable medical or administrative reasons. Possible reasons for early study termination include:

- insufficient recruitment;
- protocol violations;
- inaccurate or incomplete data;
- unethical or dangerous practices at investigational sites;
- request from Regulatory Authorities, Ethics Committee or Data and Safety Monitoring Board.

Early termination of the study must be reported to the Regulatory Authorities and Ethics Committee in accordance with local requirements.

5. Study Treatments

5.1 Administration of Study Treatment

The study treatments are nivolumab (IMP) in association with standard treatment for locally advanced cervical cancer, i.e. RT and cisplatin-based chemotherapy for 5 weeks.

Nivolumab will then be administered at the same dose level from Week 7 to Week 25. During this time, additional local treatment for cervical cancer, e.g. brachytherapy and surgery, may be performed in accordance with usual practice in each investigational center.

5.1.1 External Radiotherapy

Intensity-modulated radiation therapy (including volumetric-modulated arc therapy and tomography) will be used. A dose of 45 Gy will be delivered to the pelvis +/- para-aortic lymph nodes in 25 fractions of 1.8 Gy using a 6-MV photon energy. Para-aortic area will be treated only if tumoral involvement is documented by surgical procedure or PET. Prophylactic para-aortic radiotherapy according to institutional guidelines.

An additional dose may be delivered to invaded lymph nodes using SIB-IMRT in accordance with usual practices in each investigational center. Total dose depending on size, localization and number of lymph node will be delivered in 25 fractions (54- 57.5 Gy in 25 fractions of 2.16 to 2.3 Gy).

An additional lateral pelvic dose may be delivered if coverage of the target volumes is judged insufficient. The volumes, doses and techniques will be those usually used in each center.

The additional central pelvic dose will be delivered as brachytherapy (see Section 5.1.1) or, if this is impossible or contraindicated, as external RT, with a total dose of 64.8 Gy to 70.2 Gy.

The patient will be treated in dorsal decubitus with the usual methods of restraint in each center. For bladder distension, the patient will be given 250 ml water approximately 30 minutes prior to the RT planning computed tomography (CT) scan or RT session.

RT planning CT-scan will be performed with contrast enhancement using contiguous slices at 3 to 5-mm increments. Image fusion with diagnostic imaging (see Section 6.1 Procedures to be performed during Screening Period) may be used to assist in determining the volumes.

The clinical-anatomical target volumes for the 45-Gy dose will include the clinical target volume (CTV) of the tumor, including the gross tumor volume (GTV) with a 2-cm margin encompassing the vagina, uterine cervix and corpus. A 1-cm margin will be added to allow for of central pelvic organ motion or after defining the internal target volume (ITV) with image fusion pre- and post-bladder distension. The CTV of lymph nodes will include the obturator, internal external iliac nodes, bilateral common iliac nodes and presacral nodes.

The planned target volume (PTV) for the tumor will include the CTV with a 5 to 8-mm margin. The PTV for the 54-Gy dose to invaded lymph nodes will include a 1-cm margin.

The organs at risk will be delineated and the doses received will comply with the dose restrictions presented in Table 1.

Table 1 - Dose Restrictions for Organs At Risk

Bladder	*Pelvic +/- para-aortic 45 Gy + brachytherapy*<br>V 40 Gy<br>V 25 Gy<br>*RT alone*<br>V 70 Gy<br>V 60 Gy	< 40%<br>< 80%<br><br>< 25%<br>< 50%
Rectum	*Pelvic +/- para-aortic 45 Gy + brachytherapy*<br>V 40 Gy<br>Max. dose (posterior wall)<br>*RT alone*<br>V 74 Gy<br>V 70 Gy<br>V 60 Gy	<40%<br><25-30 Gy<br><br>< 5%<br>< 25%<br>< 50%
Femoral head	V 50 Gy<br>Max. dose	<10%<br>45-50 Gy
Small bowel	*Pelvic RT*<br>V40 Gy<br>V30 Gy<br>*Pelvic +Para aortic RT*<br>V40 Gy<br>V30 Gy	< 250 cm^3<br>< 500 cm^3<br><br>< 300 cm^3<br>< 650 cm^3
Kidney	Mean dose<br>Σ 2 kidneys	< 16 Gy<br>20 Gy < 50%
Spinal cord	Dmax	<45 Gy

Whenever possible, RT will begin on Monday.

On the days when chemotherapy is to be administered, RT will be performed 2 hours after administration of nivolumab and chemotherapy.

The total duration of RT, including brachytherapy, is not to exceed 55 days.

5.1.2 Brachytherapy

The window for brachytherapy is from Week 7 to Week 8.

Pulsed dose rate (PDR) or high dose rate (HDR) modality will be used. Each center will use the applicator of its choice. Interstitial brachytherapy may be used.

The basic principles and parameters of GYN GEC-ESTRO [15] will used to determine the target volumes and organs at risk, delineated using CT-scan and, if possible, MRI, with the applicator in place.

Target volumes are GTV, high-risk CTV and intermediate-risk CTV.

Organs at risk at the bladder, rectum and sigmoid colon.

Target volume doses and dose restrictions on organs at risk will follow GYN GEC-ESTRO.

Target volumes: HR-CTV D90= 85 Gy (EQD2; α/β10);
IR-CTV D98= 60 Gy (EQD2; α/β10).

Organs at risk: rectum, sigmoid colon: D2cc < 70 Gy (EQD2; α/β=3);
bladder: D2cc < 80-90 Gy (EQD2; α/β=3).

5.1.3 Chemotherapy

Chemotherapy, i.e. cisplatin, will be administered at 40 mg/m^2 (maximum total dose per infusion 80 mg), over 1 to 3 hours at the discretion of the local investigator, once a week throughout the external RT, and starting on Day 1 of RT. Each patient will receive 5 doses of cisplatin, except in the event of toxicity.

Cisplatin will be administered in association with intravenous hydration, in accordance with usual practice in the centers, as well as with antiemetic treatment, i.e. aprepitant and a setron. If corticosteroids are required as antiemetics, the dose is not to exceed the equivalent of 40 mg of methylprednisolone on the day of infusion of cisplatin in accordance with usual practice in the centers.

If it is necessary to discontinue cisplatin due to specific toxicity, e.g. neurotoxicity, ototoxicity or nephrotoxicity, cisplatin will be switched to carboplatin AUC 2 every week during RT.

When nivolumab and cisplatin are administered the same day, the infusion of nivolumab will take place before the infusion of cisplatin. On the days when chemotherapy is to be administered, RT will be performed 2 hours after administration of nivolumab and of chemotherapy.

5.1.4 Additional Local Treatment

Surgery for resection of residual tumor after external RT and brachytherapy is permitted, in accordance with usual practice in the investigational centers, but will not be mandatory. The window for surgery is from Week 14 to Week 16. In the event of surgery, specimens of residual tumor may be collected for ancillary studies (see Appendix 5)

5.1.5 Handling of Toxicities related to Study Treatments

Detailed information on the handling of toxicities related to RT and cisplatin-based chemotherapy is presented in Appendix 4 - Management of Toxicities related to Study Treatments.

5.1.6 Record of Administration

Administration of study treatments will be recorded in the eCRF.

5.1.7 Prohibited Concomitant Medication

All medication listed as non-inclusion criteria (see Section 4.1.2) will continue to be prohibited as concomitant treatment throughout the study. In addition, drugs known to induce hepatotoxicity should be used with caution in patients during treatment with nivolumab.

5.2 **Investigational Medicinal Product**

5.2.1 Description of the IMP

The IMP is nivolumab (BMS-936558-01). The drug product for injection is a sterile, non-pyrogenic, isotonic, aqueous solution for single use, formulated at 10 mg/ml. It is formulated in sodium citrate, sodium chloride, mannitol, diethylenetriaminepentacetic acid (pentetic acid) and polysorbate 80 (Tween® 80) at pH 6.0, and it includes an overfill to allow for vial, needle and syringe holdup.

The solution is a clear to opalescent, colorless to pale yellow liquid, which may contain a few light particulates.

Nivolumab will be supplied by Bristol-Myers Squibb.

5.2.2 Dosage Form and Packaging

Nivolumab is formulated at 100 mg/vial (10 mg/ml) in 10-cc type-1 flint glass vials, stoppered with butyl stoppers and sealed with aluminum seals.

The vials are packaged in a carton containing 5 or 10 vials.

5.2.3 Labeling

Each vial of Nivolumab will have an investigational product label permanently affixed to the outside stating that the material is for clinical trial/investigational use only. Each participating site will be in charge of labeling the cartons and vials of IMP. The labels will include the dosing instructions and a blank space for the patient identification number to be added at the time of dispensing.

5.2.4 Storage

Nivolumab must be stored between 2 and 8° Celsius, protected from light and from freezing. If the vials are stored in a glass-front refrigerator, they must be kept in the carton.

The Investigator is responsible for ensuring that the IMP is stored under the appropriate environmental conditions regarding temperature and light.
Excursions in temperature during storage must be investigated by the sponsor and managed as recommanded in the quality agreement signed between the sponsor and Bristol-Myers Squibb.

5.2.5 Dose Calculation and Administration

Nivolumab will be administered by IV infusion at a flat dose of 240 mg (or 1 mg/kg if DLT is considered unacceptable, see Section 3) over 30 minutes every 2 weeks starting on Day 1 of RT. The interval between two infusions should not be less than 12 days. When nivolumab and cisplatin are administered the same day, the infusion of nivolumab will take place before the infusion of cisplatin. On the days when chemotherapy is to be administered, RT will be performed 2 hours after administration of nivolumab and of chemotherapy.

Calculation of the dose to be administered will be based on the actual body weight at baseline. If the patient's weight on the day of dosing differs by > 10% from the weight used to calculate the original dose, the dose will be recalculated. All doses should be rounded to the nearest milligram.

There is no recommended premedication for nivolumab at the first cycle.

Nivolumab is to be administered as an IV infusion through a 0.2-micron to 1.2-micron pore size, low-protein-binding polyethersulfone membrane in-line filter at the protocol-specified dose. It is not to be administered as an IV push or bolus injection. Nivolumab can be infused undiluted (10 mg/ml) or diluted with 0.9% sodium chloride for injection, USP or 5% dextrose for injection, USP to protein concentrations as low as 1 mg/ml. Care must be taken to assure sterility of the prepared solution as the product does not contain any antimicrobial preservative or bacteriostatic agent.

Patients will be carefully monitored for infusion reactions during the administration of nivolumab and, in the event of an acute infusion reaction, the patient will be managed in accordance with usual practice in the investigational centers.

Each patient will receive 13 doses of nivolumab, except in the event of toxicity. The administration of nivolumab may be interrupted, delayed, or discontinued depending on how well the patient tolerates treatment.

5.2.6 Handling of Toxicities related to IMP

For detailed information on the handling of DLT related to nivolumab, see Section 3 Study Design.

For detailed information on the handling of other toxicities related to nivolumab, see Appendix 4 - Management of Toxicities related to Study Treatments.

5.2.7 IMP Accountability

Administration of the IMP, including dispensing and dosing, will be accurately recorded in the patient's medical file and in the appropriate section of the patient's eCRF. Vial numbers must be recorded when the IMP is dispensed.
The Investigator or a designee is responsible for taking an inventory of each shipment of IMP received, and comparing it with the accompanying IMP accountability form. The Investigator will verify the accuracy of the information on the form, sign and date it.

5.2.8 IMP Handling and Dispensing

The IMP will be delivered to each of the three investigational sites by Bristol-Myers Squibb.

The Investigator must ensure that the receipt, storage, handling, dispensing, use, returns, loss, destruction or any other disposition of the IMP are appropriately documented on the IMP accountability forms.

The Investigator in each investigational site must ensure that the IMP is dispensed only to patients participating in the study. The IMP must be dispensed only in official study sites by authorized personnel in accordance with local regulations. If concerns regarding the quality or appearance of the IMP arise, it must not be dispensed and the Sponsor must be informed without delay.

5.2.9 IMP Destruction

It is the responsibility of the Investigator to arrange for destruction/disposal of all empty IMP vials and cartons in accordance with applicable laws and regulations.

The Investigator is allowed to destroy/dispose of any unused IMP provided the following minimum conditions are met:

- on-site disposal practices do not expose humans to any risks from the IMP;
- on-site disposal practices and procedures comply with applicable laws and regulations, including any special requirements for controlled or hazardous substances;
- written procedures for on-site disposal are available and followed. The procedures must be provided to the Sponsor upon request;
- appropriate IMP accountability and disposal records are available, including the date of disposal, quantity disposed of and identification of the person responsible.
  The method of disposal, i.e. incinerator, licensed sanitary landfill, or licensed waste disposal vendor must be documented.

6. Schedule of Study Assessments and Visits

The schedule of visits and assessments are presented in Appendix 1 – Flow Chart.

The study will consist of 4 main periods:

- Screening period; from Day - 45 to Day 0
- Treatment period; from Week 1 to Week 5, nivolumab + radio-chemotherapy
  from Week 7 to Week 25, nivolumab +/- brachytherapy +/- surgery
- Follow-up period; from Week 26 to Week 104.

The study schedule in shown in Figure 1.

Figure 1. Study Schedule

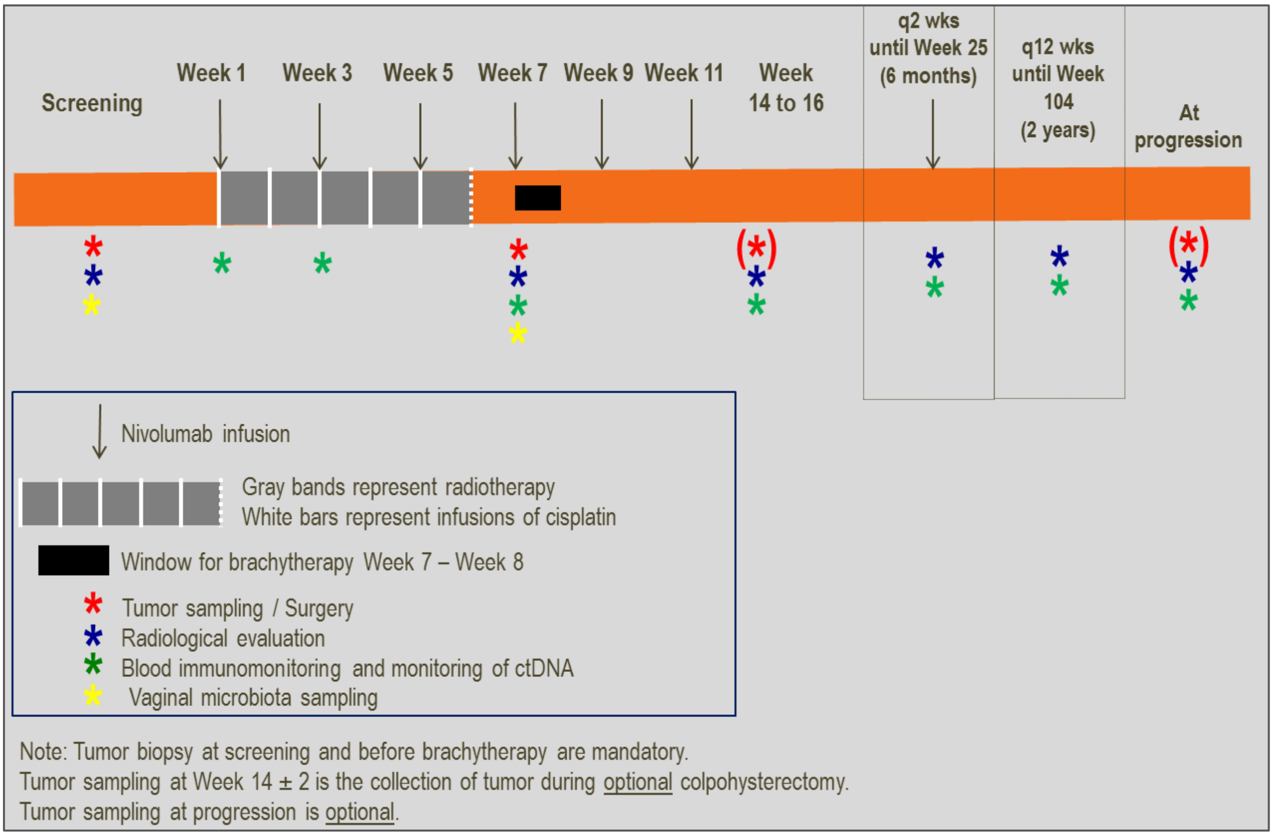

6.1 Procedures to be Performed during Screening Period

The following procedures are to be performed during the Screening Period, i.e. within 45 days before the initiation of treatment unless otherwise stated :

- Provision of information to patient and collection of informed consent;
- Assessment of inclusion and non-inclusion criteria;
- Collection of medical history;
- Collection of concomitant treatment;
- Physical examination (including height, weight and vital signs);
- Duplicate 12-lead ECG within 24 hours at least 5 minutes apart within 7 days prior to initiation of treatment;
- Collection of tumor sample and histologic assessment (see Appendix 5):
  - three tumor samples, one fresh, one formalin-fixed paraffin-embedded (FFPE) and one instantly frozen in liquid nitrogen and stored at -80°C; the minimum size required is 4-5 mm per sample. Biopsies for fresh and FFPE are a priority and the frozen fragment should not be performed if there is not enough material for FFPE (diagnosis) and fresh.
- Vaginal microbiota sampling using a swab during biopsy procedure (instantly frozen in liquid nitrogen and stored at -80°C)

- Collection of blood samples for ancillary studies:
  - 2 ml of peripheral blood on ethylene diamine tetraacetic acid (EDTA) tubes for DNA extraction and sequencing analyses;
  - 5 ml on serum dry tube with separating gel;
  - 30 ml of peripheral blood on EDTA tubes for immune analyses and monitoring of circulating tumor DNA.
- Laboratory tests, within 7 days prior to initiation of treatment, including:
  - hematology: white blood cell count, absolute neutrophil count, absolute lymphocyte count, hemoglobin, platelets;
  - blood chemistry: creatinine, AST, ALT, total bilirubin, prothrombin rate, partial thromboplastin time, SCC, LDH, CEA and CA125,….
  - serology: HBV, HCV, HIV;
- Blood or Urinary pregnancy test for women of childbearing potential*;
- Imaging for RECIST v1.1:
  - abdominal-pelvic magnetic resonance imaging (MRI) with gadolinium enhancement, T1- and diffusion-weighted sequences;
  - [^{18}F]-FDG PET-CT
- thoracic-abdominal-pelvic computed tomography (CT) scan and/or para-aortic dissection may be performed in accordance with usual practice in each investigational center and at the Investigator's discretion.

* “Women of childbearing potential” are defined as women who have experienced menarche and who have not undergone surgical sterilization (hysterectomy or bilateral oophorectomy) or who are not postmenopausal. Menopause is defined clinically as 12 months of amenorrhea in women over 45 in the absence of other physiological causes. In addition, women under the age of 62 must have a documented serum follicle stimulating hormone level < 40 mIU/ml.

6.2 Procedures to be Performed during Treatment Period

At the beginning and during the Treatment Period, patient visits will take place weekly (+/- 2 days) from Week 1 until the end of RT, and then every 2 weeks (+/- 2 days) from Week 7 to Week 25. The following procedures are to be performed:

- Physical examination (including height, weight and vital signs);
- Laboratory tests, including:
  - hematology: white blood cell count, absolute neutrophil count, absolute lymphocyte count, hemoglobin, platelets;
  - blood chemistry: creatinine, AST, ALT, total bilirubin, prothrombin rate, partial thromboplastin time, SCC, LDH, CEA and CA125,….
- Collection of concomitant treatment and adverse events;
- Radiological evaluations (the same imaging must be used for tumor evaluations);

- pelvic MRI with gadolinium enhancement, T1- and diffusion-weighted sequences; at the end of RT at Week 6, between Week 14 and Week 16 (i.e after brachytherapy and before surgery if applicable) and at Week 25.
- [^{18}F]-FDG PET-CT (recommended) or thoracic abdominal pelvic CT-scan, at Week 25.

- Vaginal microbiota sampling using a swab during brachytherapy initiation (instantly frozen in liquid nitrogen and stored at -80°C);
- Collection of tumor sample (see Appendix 5) at brachytherapy initiation, if applicable, will be done and optionally at surgery between Week 14 and Week 16 in the event of surgery for resection of residual tumor (see Section 5.1.4).

  A collection of four tumor samples (five if possible) is expected:
  - one FFPE sample,
  - one fresh sample (two if possible) for immediate analysis of the tumor microenvironment,
  - and 2 samples to be instantly frozen in liquid nitrogen and stored at -80°C.
- Collection of blood samples for ancillary studies*, only at Weeks 3, 7, 13 and 25:
  - collection of 5 ml on serum dry tube with separating gel;
  - collection of 30 ml of peripheral blood on EDTA tubes for immune analyses and monitoring of circulating tumor DNA;

  ** Note: in the event of anemia with hemoglobin > 8.0 g/dL, collection of samples for ancillary studies may be stopped until anemia resolves. Blood sample collection to monitor toxicity related to chemotherapy and nivolumab will continue.*
- Pelvic+/- para-aortic RT, during radiation period;
- Administration of cisplatin, weekly from Week 1 to Week 5; Administration of nivolumab, every 2 weeks from Week 1 to Week 25;
- Brachytherapy, less than 55 days after the start of treatment, i.e. in Week 7 or 8.

6.3 Procedures to be Performed during Follow-up Period

During the Follow-up Period, patient visits will take place every 12 weeks (+/- 5 days) up to Week 104 (2 years). The following procedures are to be performed:

- Physical examination (including height, weight and vital signs);
- Laboratory tests, including:
  - hematology: white blood cell count, absolute neutrophil count, absolute lymphocyte count, hemoglobin, platelets;
  - blood chemistry: creatinine, AST, ALT, total bilirubin, prothrombin rate, partial thromboplastin time, SCC, LDH, CEA and CA125,….
- Collection of concomitant treatment and adverse events;
- Collection of blood samples for ancillary studies:
  - collection of 5 ml on serum dry tube with separating gel;
  - collection of 30 ml of peripheral blood on EDTA tubes for immune analyses and monitoring of circulating tumor DNA;

Radiological evaluations, every 24 weeks (6 months) up to Week 104 (2 years):

- pelvic MRI with gadolinium enhancement, T1- and diffusion-weighted sequences;
- [^{18}F]-FDG PET-CT (recommended) or thoracic abdominal pelvic CT-scan.

6.4 Procedures to be Performed at End-of-study Visit

The following procedures are to be performed at the end-of-study visit at Week 104 or earlier at any time during the Treatment Period or the Follow-up Period if disease progression is detected or if end of study occurs (see section 4.3):

- Physical examination (including height, weight and vital signs);
- Laboratory tests, including:
  - hematology: white blood cell count, absolute neutrophil count, absolute lymphocyte count, hemoglobin, platelets;
  - blood chemistry: creatinine, AST, ALT, total bilirubin, prothrombin rate, partial thromboplastin time, SCC, LDH, CEA and CA125,….
- Collection of concomitant treatment and adverse events;
- Radiological evaluations in the event of disease progression:
  - pelvic MRI with gadolinium enhancement, T1- and diffusion-weighted sequences;
  - [^{18}F]-FDG PET-CT (recommended) or thoracic abdominal pelvic CT-scan.
- Collection of blood samples for ancillary studies in the event of disease progression:
  - collection of 5 ml on serum dry tube with separating gel;
  - collection of 30 ml of peripheral blood on EDTA tubes for immune analyses and monitoring of circulating tumor DNA;
- Collection of tumor sample (see Appendix 5) in case of disease progression is optional.

7. Efficacy Assessments

7.1 Response to Treatment

Assessment of the objective response rate is a secondary objective of the study.

Baseline assessments will be performed within 28 days before treatment start using MRI and CT-scan or [^{18}F]-FDG PET-CT. Imaging will include the chest, abdomen and pelvis.

Subsequent assessments should also include the chest, abdomen, pelvis, and should use the same imaging techniques as those used at baseline.

Patients will be assessed for tumor response 6 weeks (± 1 week) after treatment start, between Week 14 and Week 16 (before surgery), at Week 25 and every 24 weeks thereafter.

- Changes in tumor measurements and tumor response will be assessed by the Investigator in each investigational center using RECIST version 1.1 [14].
- Assessment of the 2-year progression-free survival rate and 2-year disease-free survival rate are also secondary objectives. Patients will be assessed by the Investigator in each investigational center every 12 weeks (± 1 week) for 2 years after end of study treatment.

8. Molecular, Tumor Microenvironment and Immune Analyses

8.1 Molecular Analyses on Tumor Samples

Retrospective exome, RNA and targeted sequencing analyses will be performed on all patients treated and for whom tumor samples are available.

Biopathology approaches will be used for the validation of molecular alterations detected by molecular analyses: exome, dedicated next-generation sequencing will be performed.

Retrospective ultra-deep sequencing to assess tumor heterogeneity may be performed on a small subset of patients in whom ctDNA monitoring at various timepoints detects *de novo* mutation.

8.2 Molecular Analyses on ctDNA

Retrospective exome and targeted sequencing analyses will be performed on all patients treated and for whom blood samples are available at the different timepoints (i.e. baseline, at Weeks 3, 6 and 12 and every 12 weeks up to Week 104) to assess temporal heterogeneity.

8.3 Analyses on Tumor Microenvironment

Detailed phenotypic analysis of the different components of the tumor microenvironment using various technologies will be used to evaluate tumor epithelial cells, endothelial cells, cancer associated fibroblast subsets and various immune cells types, i.e. T, B and NK cells, macrophages, neutrophils and DC subsets.

Systemic description of the soluble tumor microenvironment will be performed through the Luminex-based analysis of tumor-conditioned supernatants that reflect the composition of the soluble tumor microenvironment of human fresh primary tumors.

8.4 Immune Analyses

Whole-blood phenotype analysis will be performed on fresh blood samples to characterize the proportion of memory and naïve T cells, effector and memory subsets, innate-like T cells, regulatory T cells, B cells and NK cell subsets, as well as different activation markers.

Expression of PD-1/-L1 and other checkpoint molecules on the immune cells and tumor stroma will be assessed.

9. Safety Assessments

9.1 Safety

Safety will be assessed by monitoring DLT (see Section 3.3 for definitions) in the context of the primary objective of the study.

Safety will also be assessed by monitoring all AEs and abnormal laboratory findings in the context of the secondary objective of the study concerning the overall safety profile of the association of nivolumab and chemoradiation therapy.

9.2 Definition of Adverse Event

An AE is defined as any untoward medical occurrence in a patient or clinical trial subject administered a medicinal product and which does not necessarily have a causal relationship with the study treatment.

An AE can therefore be any unfavorable and unintended sign (including a clinically relevant abnormal laboratory finding or aggravation of a pre-existing condition), symptom or disease temporally associated with the use of a medicinal product, whether or not considered related to the medicinal product.

Progression of the disease, i.e. cervical cancer, will not be considered as an AE unless it leads to serious impairment.

An overdose, accidental or intentional, whether or not it is associated with an AE, or abuse, withdrawal, sensitivity or toxicity to study treatment should be reported as an AE. If an overdose is associated with an AE, the overdose and adverse event should be reported as separate terms.

An abnormal laboratory value is considered to be an AE if the abnormality:

- results in withdrawal of the patient from the study;
- results in modification of the dose of study treatment or interruption of treatment;
- requires medical treatment or any therapeutic intervention;
- suggests a disease and/or organ toxicity *and* the abnormality was not present at the baseline assessment or is considered to have worsened since the baseline assessment.

Regardless of the severity, only laboratory abnormalities that fulfill a seriousness criterion will be documented as SAEs.

If a laboratory abnormality is one component of a diagnosis or syndrome, then only the diagnosis or syndrome should be recorded in the AE section of the eCRF. If the laboratory abnormality is not part of a diagnosis or syndrome, then it should be recorded as the AE.

9.3 Definition of Serious Adverse Event

A serious adverse event (SAE) is defined as any untoward medical occurrence that at any dose :

- results in death;
- is life-threatening;
- requires in-patient hospitalization or prolongation of existing hospitalization;
- results in persistent or significant disability or incapacity;
- is a congenital anomaly or birth defect;

- constitutes an important medical event in which the patient is not at risk of death or which does not directly result in death or hospitalization, but which may jeopardize the patient or may require an intervention to prevent one of the above-mentioned outcomes.

Occurrence of a second cancer will be considered as an SAE and is to be followed 2 years after the first administration of study treatment.

All occurrences of potential Drug Induced Liver Injury (DILI) meeting the defined criteria, must be reported as SAEs.

Potential Drug Induced Liver Injury is defined as:
1. AT (ALT or AST) elevation > 3 times upper limit of normal (ULN);
AND
2. Total bilirubin > 2 times ULN, without initial findings of cholestasis (elevated serum alkaline phosphatase);
AND
3. No other immediately apparent possible causes of AT elevation and hyperbilirubinemia, including, but not limited to, viral hepatitis, pre-existing chronic or acute liver disease, or the administration of other drug(s) known to be hepatotoxic.

The following events will not be considered as SAEs:

- hospitalization < 24 hours;
- elective hospitalization

9.4 Definition of Adverse Reaction

An adverse reaction is any untoward and unintended response to an investigational medicinal product related to any dose administered, which means that there is at least a possible relationship between administration of the IMP and the adverse reaction.

9.5 Definition of Suspected Unexpected Serious Adverse Reaction

A SUSAR is an event that is not mentioned in the Nivolumab Investigator Brochure or in the cisplatin SPC or that is not consistent with the information in the Investigator Brochure as concerns the nature, severity, outcome or frequency of the SAE.

9.6 Severity Criteria

Severity is not to be confused with seriousness, which is used to define the requirements in terms of reporting.

The severity of an AE is to be assessed according to the NCI CTC-AE, version 4.03. If the severity of any AE is not listed in the NCI CTC-AE, the following scale will be used:

Mild (grade 1)............................ does not affect the patient's usual daily activity
Moderate (grade 2)................... disrupts the patient's usual daily activity
Severe (grade 3)........................ precludes the patient's usual daily activity
Life-threatening (grade 4) .... requires intensive care / is life-threatening
Fatal (grade 5) .... results in death

9.7 Methods and Timeframes for Collecting Safety Data

Serial safety assessments will be performed at scheduled intervals throughout the study as outlined in Appendix 1 : Flow-Chart.

The periods for collection of safety data, i.e. DLT and AEs that occur in the context of the study are defined as follows:

- **for any DLT**: from the time of first administration of a study treatment until the visit at Week 12;
- **for any SAE**: from the time of enrolment of the patient in the study (after signature of the informed consent form) until 100 days after the last intake of the IMP (i.e. second follow-up visit);
- **for any AE** that is not considered to be an SAE: from the time of first administration of a study treatment until 100 days after the last intake of the IMP (i.e. second follow-up visit);

AEs considered to be possibly related to study participation that occur during the Screening Period, i.e. after signature of the informed consent form and before the first administration of a study treatment, will also be collected and recorded in the e-CRF.

Safety data will be routinely collected at each visit by performing physical examinations and laboratory tests, as well as by questioning the patient directly (solicited reports) and indirectly (spontaneous reports) using a question such as “How have you been feeling?” or “Have you experienced any adverse events since the last visit?”.

All AEs must be recorded in the “Adverse Events” section of the e-CRF.

Safety data must be reviewed by the Investigator. The Investigator must determine whether the AE is considered to be serious or not, as this distinction will determine the procedure for reporting the event. The Investigator must also determine whether the AE or SAE is possibly related to the study treatment.

9.8 Reporting SAEs

All SAEs and SUSARs, whether or not they are considered to be related to the study treatment, that occur during the SAE collection period defined in Section 9.7 (except for second cancer, for which the collection period is 5 years), must be reported to the R&D Unicancer Safety Department.

Any SAE that occurs after the SAE collection period defined in Section 9.7 must also be reported to the R&D Unicancer Safety Department if the Investigator considers that it is possibly related to the study treatment.

The SAE report must be sent by fax to the R&D Unicancer Safety Department within 24 working hours after the Investigator becomes aware of the event using the SAE reporting form**:**

R&D Unicancer – Pharmacovigilance
Tel.: 01 44 23 04 16 – Fax: 01 44 23 55 70
Email: pv-rd@unicancer.fr

Additional information may be requested by the R&D Unicancer Safety Department or by the Clinical Research Associate through an SAE Query Form sent to the investigator site.

For each event, the Investigator must report:
- the patient ID number;
- the nature of the event, as clearly as possible using appropriate medical terminology;
- the severity;
- the start date and end date of the event;
- the seriousness criterion;
- the measures taken, including corrective treatment;
- status of the study treatment, i.e. dose reduction or temporary discontinuation;
- the outcome;
- the relationship to the study schedule or to a protocol procedure, e.g. treatment regimen, further investigations specifically requested by the protocol;
- the relationship to the study treatment, to the disease under study, to concurrent disease or to concomitant treatment.

Whenever possible, the Investigator should also provide:
- a copy of the hospitalization report or report on prolongation of hospitalization;
- a copy of the autopsy report;
- a copy of all laboratory test results, including relevant negative findings (with normal laboratory values);
- any other document that is relevant in the Investigator's opinion.

All these documents must be rendered anonymous. Additional information may be requested by the R&D Unicancer Safety Department or the study CRA.

9.9 Serious Adverse Event Follow-up

The Investigator is responsible for appropriate medical follow-up of patients who experience SAEs until recovery, stabilization or death of the patient. If necessary, SAE follow-up may continue beyond the end of the study.

The Investigator will provide additional information to the R&D Unicancer Safety Department using an SAE reporting form within 48 hours. The box "Follow-up" must be ticked to indicate that it is not an initial report. The final follow-up form is to be sent after recovery, stabilization or death of the patient.

The Investigator will retain all documents regarding the SAE in order to respond to any requests for further information from the R&D Unicancer Safety Department.

10. Statistical Considerations

10.1 Sample Size and Power Considerations

The minimum sample size will be 15 evaluable patients and the maximum will be 21 evaluable patients. Patients who failed to complete the first 11 weeks of treatment (= DLT evaluation period) for a reason other than DLT, or who received a reduced dose of study drugs (<70% of the planned dose) for a reason other than DLT, will be considered as not evaluable for the primary toxicity endpoint assessment.Non-evaluable patients should be replaced.

The study will use a 3+3 design to confirm the dose of nivolumab, i.e. 240-mg flat dose q2wk. The first cohort of 3 patients will receive nivolumab at 240-mg flat dose q2wk in association with chemoradiation therapy from Week 1 to week 5 and nivolumab alone (+/- brachytherapy) from Week 7 to Week 11 and, if DLT is considered acceptable, i.e. present in 1 or 0 patients, an additional cohort of 3 patients will be included until 6 patients have been treated. If DLT is considered unacceptable, i.e. present in 2 or 3 patients, this first cohort will be stopped and a second cohort of 3 patients will be set up. In this second cohort the 3 patients will receive nivolumab at 1 mg/kg q2wk in combination with chemoradiation therapy from Week 1 to Week 5 and nivolumab alone (+/- brachytherapy) from Week 7 to Week 11 and, if DLT is considered acceptable, i.e. present in 1 or 0 patients, an additional cohort of 3 patients will be included until 6 patients have been treated. If DLT is considered unacceptable, i.e. present in 2 or 3 patients, the second cohort will be stopped and the study will terminate.

Thus, the DLT assessment phase will include up to 6 patients at the given dose. DLT will be considered acceptable if 1 or 0 patients among the first 6 DLT evaluable patients develop DLT.

An expansion cohort of 9 patients will then open to collect additional data on the safety profile of nivolumab in association with radio-chemotherapy and as adjuvant therapy in the treatment of locally advanced cervical cancer.

10.2 **Statistical Analysis**

10.2.1 Primary Safety Analysis

The Phase I main analysis set will include only patients deemed evaluable for the DLT evaluation (DLT population). Patients who failed to complete the first 11 weeks of treatment (= DLT evaluation period) for a reason other than DLT, or who received a reduced dose of study drugs (<70% of the planned dose of Nivolumab/chemotherapy/radiotherapy) for a reason other than DLT, will be considered as not evaluable for the primary toxicity endpoint assessment.

Safety analyses will include:

- Maximum CTCAE grade (Version **CTCAE v4.03**) for adverse events;
- Serious adverse events during the treatment period and during DLT period;
- Study discontinuation due to adverse event;
- Death occurring during the study treatment period and during DLT period.

10.2.2 Analyses of Secondary Efficacy and Safety Endpoints

The objective response rate, progression-free survival rate, and disease-free survival rate will be the secondary endpoints. The analyses will be exploratory and descriptive, and will be performed on all patients who received at least one administration of the recommended dose of nivolumab, whether or not they are evaluable for DLT, in order not to overestimate the response rate or long-term criteria as progression-free survival, by excluding patients who progress or die during the DLT window.

The objective response rate (ORR) is defined as the proportion of all subjects whose best response is either a complete response or a partial response, as assessed using RECIST v1.1, in order to

determine the best overall response from the baseline measurements.

The progression-free survival rate (PFS) is defined as the duration from start of the treatment to disease progression or death, regardless of cause of death.

The disease-free survival rate (DFS) is defined as the duration from the start of complete response to the time of relapse from complete response. DFS applies only to patients in complete response.

The ORR will be given with 95% exact confidence interval with binomial distribution. PFS and DFS will be estimated by the Kaplan-Meier method and given with their 95% confidence interval.

11. Data Protection and Handling of Confidentiality

Until the study results are published, the investigator is responsible for ensuring the confidentiality of the totality of the information, handled by her/him and all other individuals involved in the conduct of the study. This obligation holds does not apply to the information that the investigator must communicate to the patients within the context of the study or to information that is already published.

Nevertheless, in conformity with Article R 5121-13 of the Public Health Code, both the center and the investigator may communicate information relative to the study to:

- the Ministry of Health;
- public health inspectors;
- the General Director of ANSM and inspectors.

All study data will be processed in accordance with the methodology of Reference 001 published by the French national commission on data protection (CNIL). Institut Curie has ensured compliance with this methodology before submitting the study to the Ethics Committee and ANSM.

12. Quality Assurance and Quality Control

In order to ensure the validity, accuracy and completeness of the data in accordance with Good Clinical Practices, the Sponsor will set up a quality assurance program including:

- management of the study according to the Institut Curie procedures;
- quality control on the data provided by the investigational site, performed by the study monitor whose role is to carry out source document verification with regard to data recorded in the eCRF;
- possible audits of investigational sites.

13. Ethical and Regulatory Considerations

The study will be conducted in accordance with:

- the ethical principles set forth in the most recent version of the Declaration of Helsinki;
- Good Clinical Practices, as defined by the International Conference on Harmonization (ICH–E6, 17/07/96 and intergrated addendum);

- European Directive 2001/20/CE on the conduct of clinical trials;
- The law on 'data files and freedom' (Informatique et Libertés n° 78-17) of January 6, 1978 amended notably by Law n° 2016-41 of January 26 2016 on modernization of the National Health System and by law n° 2018-493 of June 20th 2018 relating to personal data protection;
- Bioethics Law n° 2004-800 of August 6 2004;
- Law n° 2012-300 of March 5 2012 related to the research involving human person and its implementing legislation.
- Regulation of the European Parliament and of the Council of 27 April 2016 on the protection of natural persons with regard to the processing of personal data and on the free movement of such data.

13.1 Ethics Committee (CPP)

Prior to the study start, the Sponsor will submit the protocol for opinion from one of the competent Ethics Committee (CPP) in the region where the principal investigator is practicing.

A favorable opinion must be received from the CPP prior to starting the study.

Requests for substantial modifications to the protocol, i.e. amendments, if any, will also be submitted for opinion from the CPP prior to implementation.

13.2 Competent Authority

Prior to the study start, the Sponsor will submit the protocol for approval from the competent authority, i.e. the National Agency for the Safety of Medicines and Healthcare Products (ANSM) in France.

Approval from ANSM must be received prior to starting the study.

Requests for substantial modifications to the protocol, i.e. amendments, if any, will also be submitted to ANSM for approval prior to implementation.

13.3 Information and Consent of Participants

Prior to performing any study-specific procedure, written informed consent must be obtained from each individual participating in the study after she has been informed by the investigator during a physician-patient consult and after the patient has been given sufficient time for reflection. Informed consent will be documented by signature of the Informed Consent Form.

Information given to the trial participants must cover all of the elements defined by the law n° 2012-300 of March 5th, 2012 relating to the researches involving the human person and must be written in a simple and comprehensible patient-appropriate manner. Once the participant is acquainted with the information, she/he must sign the information sheet. The original sheet will be kept in the investigator's folder and the duplicate copy will be returned to the participant.
The Informed Consent Form will be dated and signed by both the participant and the Investigator. The original form will be kept in the investigator site file and a copy will be given to the participant.

The consent form must be dated and signed by both the participant in research and the investigator. The original document is archived by the investigator; a copy is given to the research participant.

The Patient Information and Informed Consent Form must be in the same document to ensure that the all relevant information is provided to the participant.

13.4 Responsibilities of the Sponsor

The Sponsor, i.e. Institut Curie, of the study is the natural or moral person that takes the initiative of conducting research on human subjects, and is therefore accountable for the management of the study and for verifying the financing schedule.

The main responsibilities of the Sponsor are:

- to contract insurance for civil liability;
- to register the study in the ANSM database;
- to request the opinion of the Committee for the Protection of Persons (CPP) on the initial project and the substantial amendments;
- to submit an application for clinical trial authorization from the competent authority and to inform them of any substantial amendments;
- to provide information on the study to the heads of the healthcare centers, the investigators and the pharmacists;
- to report any SUSARs related to any of the study treatments to the competent authorities, i.e. ANSM and EMEA (via the European pharmacovigilance database Eudravigilance) and to communicate the information to the CPP and Investigators;
- preparation of the annual development safety update report and submission to the competent authority and the CPP;
- the declaration of the beginning and the end of the study to the competent authority,
- preparation of the final study report;
- communicating information on the results of the study to the competent authority, the CPP and the research participants;
- archiving the essential study documents in the Sponsor file for a minimum of 15 years after the end of the study.

13.5 Responsibilities of the Investigator

The Investigator in each of the investigational centers agrees to conduct the clinical study in accordance with the protocol that has been approved by the CPP and the competent authority.

The Investigator must not introduce any change to the protocol without having obtained prior written authorization from the Sponsor. The CPP and the competent authority must approve the proposed modifications prior to implementation.

It is the responsibility of the Principal Investigator:

- to provide the Sponsor with an up-to-date curriculum vitae, and with those of any co-investigators;
- to identify the members of the team that will participate in the study and to define their responsibilities;

- to ensure patient recruitment.

It is the responsibility of each Investigator:

- to collect the informed consent form, dated and personally signed by each potential study participant before any study-specific procedure is undertaken;
- to complete, on a regular on-going basis, the eCRF for each patient in the study and to ensure that the clinical research assistant mandated by the Sponsor has direct access to source documents in order to validate the data entered in the eCRF;
- to respond to any requests for data clarification in a timely manner;
- to accept regular visits of the study monitor and possibly the auditors mandated by the Sponsor or the inspectors of the competent authorities.

13.6 Federation of Patient Committees for Clinical Research in Oncology

The Federation of Patient Committees for Clinical Research in Oncology (FCPRCC) was set up on the initiative of Unicancer and the LNCC (National Anticancer League). Its dedicated task is to provide a second reading of study protocols in oncology. The FCPRCC is coordinated by the BECT (Office of Clinical and Therapeutic Studies), a department of Unicancer. It brings together patient committees from the LNCC and other healthcare centers. It reviews the protocol and proposes improvements dealing principally with the quality of the information sheet for patients and organization of the treatment and monitoring plan by suggesting measures to improve the comfort of patients.

14. Data Review and Data Management

Data analysis will be performed by the Biostatistics department of Institut Curie under the supervision of Alexia Savignoni, MD and Doctor Emanuela Romano, the study coordinator.

In accordance with Article R 5121-13 of the Public Health Code, both the center and the investigator may communicate information relative to the study:

- to the Health Minister;
- to the public health inspectors;
- to the General Director of ANSM and inspectors.

Prior to publication of the study results, the Investigator is responsible for ensuring that all information relative to the study, handled by her/him or by other individuals involved in the study, remain strictly confidential. This does not apply to information the Investigator must provide to patients in the context of the study or to information that is already publicly available.

15. Financial Information and Study Insurance

The study is financially supported by Bristol-Myers Squibb, who also agreed to provide nivolumab to the Sponsor. Notwithstanding support from Bristol-Myers Squibb, Institut Curie takes full responsibility for conducting the study and has contracted specific insurance for civil liability.

16. Publication of Results

All information relative to the study is considered to be confidential, at least until first public communication and after the information has been appropriately analyzed and checked by the Sponsor, the Principal Investigator and the Statistician of the study.

Any subsequent publication, abstract or presentation concerning the study results must be submitted for examination and approval to the Sponsor (Institut Curie).

The lead authors of the publication will be the Principal Investigator and the Coordinator of the writing committee. They may however designate another person to (co-) write the publication.

The other investigators will appear in the list of co-authors in decreasing order, according to the number of patients recruited, regardless of the importance of the cooperating group to which they belong, followed by a person representing each cooperating group among the investigational centers with the highest recruitment rates. The Statistician will be cited as a co-author. A representative of the Sponsor will be acknowledged.

In an equal manner, publication of the sub-studies (biological studies) will make mention of the name of the person who carried out the sub-studies as well as the names of all individuals who took part in carrying out these sub-studies. A representative of the Sponsor will be acknowledged.

17. References

1. Guerin S, Hill C. [Cancer epidemiology in France in 2010, comparison with the USA]. Bulletin du cancer. 2010 Jan;97(1):47-54.
2. Piersma SJ, Jordanova ES, van Poelgeest MI, Kwappenberg KM, van der Hulst JM, Drijfhout JW et al. High number of intraepithelial CD8+ tumor-infiltrating lymphocytes is associated with the absence of lymph node metastases in patients with large early-stage cervical cancer. Cancer Res. 2007;67:354–361.
3. Steele JC, Mann CH, Rookes S, Rollason T, Murphy D, Freeth MG et al. T-cell responses to human papillomavirus type 16 among women with different grades of cervical neoplasia. Br J Cancer. 2005 Jul 25;93(2):248-59.
4. van Seters M, van Beurden M, ten Kate FJ, Beckmann I, Ewing PC, Eijkemans MJ, et al. Treatment of vulvar intraepithelial neoplasia with topical imiquimod. N Engl J Med. 2008 Apr 3;358(14):1465-73.
5. Karim R, Jordanova ES, Piersma SJ, Kenter GG, Chen L, Boer JM, et al. Tumor-expressed B7-H1 and B7-DC in relation to PD-1+ T-cell infiltration and survival of patients with cervical carcinoma. Clin Cancer Res. 2009 Oct 15;15(20):6341-7.
6. Grosso J, Inzunza D, Wu Q, Simon J, Singh P, Zhang X, et al. Programmed death-ligand 1 (PD-L1) expression in various tumor types. Journal for ImmunoTherapy of Cancer. 2013;1(Suppl 1):53.
7. Postow MA, Callahan MK, Barker CA, Yamada Y, Yuan J, Kitano S, et al. Immunologic correlates of the abscopal effect in a patient with melanoma. N Engl J Med. 2012 Mar 8;366(10):925-31.
8. Postow MA, Callahan MK, Barker CA, Yamada Y, Yuan J, Kitano S et al. Immunologic correlates of the abscopal effect in a patient with melanoma. N Engl J Med. 2012 Mar 8;366(10):925-31.
9. Sharabi A, Nirschl C, Ceccato T, Nirschl T, Francica B, Alme A et al. Role of Radiation Therapy in Inducing Antigen Specific Antitumor Immune Responses When Combined With Anti-PD1 Checkpoint Blockade: Mechanism and Clinical Implications. International Journal of Radiation Oncology Biology Physics. 2014;1(Suppl 1) vol 90, Page S1.
10. Park SS, Dong H, Zhao W, Grams MP, Liu X, Harrington SM et al. PD-1 Blockade Enhances Radiation Therapy-Induced Abscopal Effect. International Journal of Radiation Oncology Biology Physics. 2014;1(Suppl 1) vol 90, Page S57.
11. Deng L, Liang H, Burnette B, Beckett M, Darga T, Weichselbaum RR, et al. Irradiation and anti-PD-L1 treatment synergistically promote antitumor immunity in mice. The Journal of clinical investigation. 2014 Feb 3;124(2):687-95.
12. CCCMAC, Cochrane Database Syst Rev. 2010 Jan 20;(1):CD008285)
13.Gerber DE, Urbanic JJ, Langer C, Lu C, Movsas B, Jeraj J, et al. Treatment Design and Rationale for a Randomized Trial of Cisplatin and Etoposide Plus Thoracic Radiotherapy Followed by Nivolumab or Placebo for Locally Advanced Non-Small-Cell Lung Cancer (RTOG 3505).Clinical Lung Cancer. 2016 Oct 26; doi: 10.1016/j.cllc.2016.10.009.
14. Eisenhauer EA, Therasse P, Bogaerts J, et al. New response evaluation criteria in solid tumours: revised RECIST guideline (version 1.1). Eur J Cancer. 2009;45:228-47.
15. Dimopoulos JC, Petrow P, Tanderup K, Petric P, Berger D, Kirisits C, et al. Recommendations from Gynaecological (GYN) GEC-ESTRO Working Group (IV): Basic principles and parameters for MR imaging within the frame of image based adaptive cervix cancer brachytherapy.Radiother Oncol. 2012 Apr;103(1):113-22.

Ensemble, prenons
le cancer de vitesse.

18. Appendices

Appendix 1: Study Flow Chart
Appendix 2: ECOG Performance Status Scale
Appendix 3: Toxicity Criteria (NCI CTC-AE)
Appendix 4: Management of Toxicities related to Study Treatments
Appendix 5: Procedures for Tumor Sampling

Appendix 1 – Flow Chart

	Screening Period		Treatment period			Follow-up Period	If disease progression
Study Procedure↓ Timeframe→	Within 45 days before registration	Within 7 days before starting treatment	From Week 1 to Week 5	From Week 7 to Week 9	From Week 11 to Week 25 (6 months)	Until Week 104 (2 years)	At any time
Patient information & informed consent	x						
Inclusion & non-inclusion criteria	x						
Medical history	x						
Physical examination		x	x (every week)	x (every 2 weeks)	x (every 2 weeks)	x (every 12 weeks)	x
Hematology and blood chemistry		x	x (every week)	x (every 2 weeks)	x (every 2 weeks)	x (every 12 weeks)	x
Duplicate 12-lead ECG		x					
Urinary pregnancy test	x						
Concomitant medication	x		x (every week)	x (every 2 weeks)	x (every 2 weeks)	x (every 12 weeks)	x
Tumor collection	x			x (at brachytherapy initiation)	x (optional; if surgery)		x (optional)
Blood sampling for ancillary studies		x	x (at Week 3)	x (at brachytherapy initiation or at Week 7)	x (at Week 13 & 25)	x (every 12 weeks)	x
Vaginal microbiota sampling		x		x (at brachytherapy initiation)			
**Study treatment**							
Radiotherapy			x				
Cisplatin-based chemotherapy			x				
Administration of nivolumab			X (every 2 weeks)	x (every 2 weeks)	x (every 2 weeks)		
Brachytherapy				x[1]			
Surgery					x[1]		
**Safety assessment**							
Dose-limiting toxicity			x	x			
Adverse events			x	x	x	x[2]	x
Serious adverse events			x	x	x	x[2]	x
**Efficacy assessment**							
Radiological assessment for RECISTv1.1	x			x (at brachytherapy initiation or at Week 7)	x (Week 14 to Week 16 (before surgery) and at Week 25)	x (every 24 weeks)	x

[1] Window for brachytherapy is Week 7 to Week 8. Window for surgery is Week 14 to Week 16. Brachytherapy and surgery will be performed according to institutional guidelines.
[2]: SAE and AE: until 100 days after the last intake of IMP.

institutCurie

Ensemble, prenons le cancer de vitesse.

Appendix 2 – ECOG Performance Status Scale

Score	Description
**0**	Fully active, able to carry on all pre-disease performance without restriction.
**1**	Restricted in physically strenuous activity but ambulatory and able to carry out work of a light or sedentary nature, e.g., light house work, office work.
**2**	Ambulatory and capable of all self-care but unable to carry out any work activities. Up and about more than 50% of waking hours.
**3**	Capable of only limited self-care, confined to bed or chair more than 50% of waking hours.
**4**	Completely disabled. Cannot carry on any self-care. Totally confined to bed or chair.
**5**	Dead.

Appendix 3 – Toxicity Criteria (NCI CTC-AE)

http://ctep.cancer.gov/

NCI Common Terminology Criteria for Adverse Events v4.03 (NCI CTC-AE)

Appendix 4 - Management of Toxicities related to Study Treatments

Toxicity	Radiotherapy	Cisplatin-based Chemo	Nivolumab
**Allergic reaction**			
Grade 2	No change	Dosing at investigator's discretion, corticosteroids allowed	Dosing at investigator's discretion, corticosteroids allowed
Grade ≥ 3	At investigator's discretion	Permanently discontinue	Permanently discontinue
**Hematological toxicity**			
Hb < 8.0 g/L	No change	-Skip dosing until resolved<br>-Transfusion and erytropoetin allowed	No change
Neutrophils < 1000/mm^3	No change	-Skip dosing until resolved<br>-G-CSF allowed for next chemotherapy injections	No change
Febrile neutropenia	Delay until resolved	-Skip dosing until apyrexia and neutrophils ≥ 1000/mm^3<br>-Treat following local guidelines<br>-G-CSF allowed for next chemotherapy dosing	
Platelets ≤ 75,000/mm^3	At investigator's discretion	Skip dosing	No change
Platelets ≤ 25,000/mm^3	Hold until platelets > 20,000/mm^3	-Skip dosing<br>-Transfusion allowed	
**Hepatic toxicity**			
Grade 1 (AST or ALT > ULN to 3.0 x ULN and/or total bilirubin > ULN to 1.5 x ULN)	No change	No change	No change
Grade 2 (AST or ALT > 3.0 to 5 x ULN and/or total blirubin > 1.5 to 3.0 x ULN)	No change	-Skip dosings until resolved to grade 1<br>-Monitor liver function twice a week<br>-0.5 to 1 mg/kg/day methylprednisolone IV or PO equivalent until grade 1, then taper steroids over at least 1 month	
Grade 3-4 (AST or ALT > 5x ULN and/or total bilirubin > 3.0 x ULN)	At investigator's discretion	-Permanently discontinue<br>-Monitor liver function every other day<br>-1 to 2 mg/kg/day methylprednisolone IV or IV equivalent until grade 1, then taper steroids over at least 1 month<br>-If persists > 3-5 days, worsens, or rebounds: consider mycophenolate mofetil 1g twice daily (if no contraindications); consult gastroenterologist	

institutCurie

Ensemble, prenons le cancer de vitesse.

Toxicity	Radiotherapy	Cisplatin-based Chemo	Nivolumab
**Pulmonary toxicity**			
Grade 1 (radiographic changes only)	At investigator's discretion	At investigator's discretion	- Consider delay (at investigator's discretion). Re-image at least every 3 weeks.<br>If worsens: Treat as Grade 2 or 3-4
Grade 2 (mild to moderate new symptoms)	At investigator's discretion	At investigator's discretion	- Delay Nivolumab (at investigator's discretion)<br>Pulmonary and ID consults<br>- Monitor symptoms daily. Re-image every 1-3 days, consider hospitalization.<br>- 1 mg/kg/day methylprednisolone IV or PO equivalent until grade 1,<br>then taper steroids over at least 1 month.<br>- If not improving after 2 weeks or worsening : treat as grade 3-4<br>- Consider bronchoscopy, lung biopsy
Grade 3-4 (severe new symptoms; new/worsening hypoxia; life-threatening	At investigator's discretion	Permanently discontinue	- Permanently discontinue Nivolumab. Hospitalize<br>Pulmonary and ID consults.<br>- 2 to 4 mg/kg/day methylprednisolone IV or IV equivalent.<br>- If improve to baseline : taper steroids over at least 6 weeks. If not improving after 48h or worsening, add additional immunosupression.<br>- Consider bronchoscopy, lung biopsy
**Renal toxicity**			
Creatinine clearance < 50mL/mn according to MDRD formula	No change	Switch to Carboplatin (AUC2) q1w	No change except if creatinine doubled compared to baseline. If so treat with 0.5 to 1 mg/kg/day methylprednisolone IV or PO equivalent then taper steroids over at least 1 month
Creatinine clearance < 40mL/mn according to MDRD formula	At investigator's discretion	-Permanently discontinue<br>- Consult nephrologist within 48hrs<br>-Consider 1 to 2 mg/kg/day prednisone equivalents then taper steroids over at least 1 month	
**Peripheral motor or sensory neuropathy**			
Grade 1 (asymptomatic; clinical or diagnostic observation)	No change	Switch to Carboplatin (AUC2) q1w	No change
Grade 2 (moderate symptoms; limiting instrumental activities of daily life)	No change	Permanently discontinue	-Skip dosing until resolved to grade 1<br>-0.5 to 1 mg/kg/day methylprednisolone IV or PO equivalent until grade 1, then taper steroids over at least 1 month
Grade ≥ 3 (severe symptoms; intervention indicated)	At investigator's discretion	Permanently discontinue	-Permanently discontinue<br>-1 to 2 mg/kg/day methylprednisolone IV or IV equivalent until grade 1, then taper steroids over at least 1 month

institutCurie
Ensemble, prenons le cancer de vitesse.

Toxicity	Radiotherapy	Cisplatin-based Chemo	Nivolumab
**Other immune-mediated toxicities**			
Grade 1	No change	No change	Monitor and inform sponsor
Grade 2	At investigator's discretion	At investigator's discretion	-Skip dosing until resolved to grade 1<br>-if symptoms persists 1 week: 0.5 to 1 mg/kg/day methylprednisolone IV or PO equivalent until grade 1, then taper steroids over at least 1 month
Grade 3	At investigator's discretion	At investigator's discretion	-Skip dosing until resolved to grade 1.<br>-1 to 2 mg/kg/day methylprednisolone IV or IV equivalent until grade 1, then taper steroids over at least 1 month
Grade 4	At investigator's discretion	- Permanently discontinue<br>-1 to 2 mg/kg/day methylprednisolone IV or IV equivalent until grade 1, then taper steroids over at least 1 month	- Permanently discontinue<br>-1 to 2 mg/kg/day methylprednisolone IV or IV equivalent until grade 1, then taper steroids over at least 1 month
**Important Note:** When administering a long course of steroids (i.e.: 1 month or longer), always consider prophylactic antibiotics for opportunistic infections.			

Appendix 5 - Procedures for Tumor Sampling

In the NiCOL study, tumor biopsies will be collected by the medical team as part of routine patient care.
Whenever possible, tumor sampling will be performed during a diagnostic or therapeutic procedure.
Specific tumor sampling will be performed for the NiCOL study only when this is not possible.
Tumor samples will be used above all for diagnostic purposes rather than for research purposes.
If the Investigator considers that the quantity of tumor material that could be taken for the study is not sufficient, tumor samples will be collected as follows.

- Preferably, a fresh sample (two if possible) will be taken and formalin-fixed, paraffin-embedded samples will be prepared;
- Alternatively, frozen tissue will be taken, depending on the size of the biopsy.

Surgical specimens are usually of sufficient size for the collection of fresh and frozen material;

If there are multiple disease sites, e.g. for tumor sampling at disease progression, the Investigator will decide which site should be selected for tumor biopsy, based on the following rules:

- A tissue sample will be collected for the study, if possible, when biopsy is necessary to confirm the diagnosis of metastatic disease (e.g. one single metastatic site, no radiological evidence);
- A tissue sample will be collected for the study, if possible, when brachytherapy will be initiated;
- A tissue sample will be collected for the study, if possible from the surgical specimen when surgery is performed for therapeutic purposes, e.g. excision of a solitary brain metastasis, cementoplasty, bone fracture or decompression due to raised intracranial pressure, medullary compression, etc.
- For patients with multiple metastatic sites, the most accessible and the least dangerous sites for the patient will be chosen for metastatic tumor sampling.
- For bone metastasis, no tumor sampling will be allowed.
- For brain metastasis, no tumor sampling will be allowed, except in the event of surgery for therapeutic or diagnostic purposes.

If the patient is receiving an anticoagulant or a platelet aggregation inhibitor, the end of the study will be decided by patient's doctor or cardiologist.
Patients receiving anticoagulants or platelet aggregation inhibitors with vitamin-K antagonists will be switched to subcutaneous anticoagulants at effective dose. These should be stopped 24 hours before biopsy and reintroduced 48 hours after. If the switch is not possible, biopsy will not be performed.
In patients receiving anticoagulants by subcutaneous injection, treatment should be discontinued 24 hours before biopsy and reintroduced 48 hours after. If discontinuation is not possible, biopsy will not be performed.
If the patient takes a platelet aggregation inhibitor, in particular, aspirin > 160 mg/day or clopidogrel, depending on the indication, the treatment should be discontinued 5 days before biopsy and reintroduced 24 hours after, or it should be switched to a subcutaneous anticoagulant, as described above. If discontinuation of the platelet aggregation inhibitor or switch to a subcutaneous anticoagulant is not possible, biopsy will not be performed.
Treatment discontinuation and/or switch must be clearly recorded in the patient's medical file.
