# Supplementary material for: Nivolumab plus chemoradiotherapy in locally-advanced cervical cancer: the NICOL phase 1 trial
Source: Nat Commun. 2023 Jun 22;14:3698. doi: 10.1038/s41467-023-39383-8 (PMC10287640; doi:10.1038/s41467-023-39383-8)
Supplement: Supplementary file 3 — Description of Additional Supplementary Files [file 41467_2023_39383_MOESM3_ESM.pdf]

Description of Additional Supplementary Files

Supplementary Data 1: List of the genes that are enriched in the pathways of the GSEA analysis, relative to Figure 2C-D.
