# Supplementary material for: Nivolumab plus chemoradiotherapy in locally-advanced cervical cancer: the NICOL phase 1 trial
Source: Nat Commun. 2023 Jun 22;14:3698. doi: 10.1038/s41467-023-39383-8 (PMC10287640; doi:10.1038/s41467-023-39383-8)
Supplement: Supplementary file 5 — Reporting Summary [file 41467_2023_39383_MOESM5_ESM.pdf]

nature portfolio

Corresponding author(s): Emanuela Romano

Last updated by author(s): Jun 11, 2023

Reporting Summary

Nature Portfolio wishes to improve the reproducibility of the work that we publish. This form provides structure for consistency and transparency in reporting. For further information on Nature Portfolio policies, see our Editorial Policies and the Editorial Policy Checklist.

Statistics

For all statistical analyses, confirm that the following items are present in the figure legend, table legend, main text, or Methods section.

n/a	Confirmed	
☐	☒	The exact sample size (*n*) for each experimental group/condition, given as a discrete number and unit of measurement
☐	☒	A statement on whether measurements were taken from distinct samples or whether the same sample was measured repeatedly
☐	☒	The statistical test(s) used AND whether they are one- or two-sided<br>*Only common tests should be described solely by name; describe more complex techniques in the Methods section.*
☒	☐	A description of all covariates tested
☐	☒	A description of any assumptions or corrections, such as tests of normality and adjustment for multiple comparisons
☐	☒	A full description of the statistical parameters including central tendency (e.g. means) or other basic estimates (e.g. regression coefficient) AND variation (e.g. standard deviation) or associated estimates of uncertainty (e.g. confidence intervals)
☐	☒	For null hypothesis testing, the test statistic (e.g. *F*, *t*, *r*) with confidence intervals, effect sizes, degrees of freedom and *P* value noted<br>*Give P values as exact values whenever suitable.*
☒	☐	For Bayesian analysis, information on the choice of priors and Markov chain Monte Carlo settings
☒	☐	For hierarchical and complex designs, identification of the appropriate level for tests and full reporting of outcomes
☒	☐	Estimates of effect sizes (e.g. Cohen's *d*, Pearson's *r*), indicating how they were calculated

Our web collection on statistics for biologists contains articles on many of the points above.

Software and code

Policy information about availability of computer code

Data collection	No software was used. All NGS, Bulk RNA-seq and experimental data were collected and generated according to in-house pipelines and methods, detailed in the methods section.
Data analysis	Multiplex-IHC:<br>Vectra (v3.0.5)<br>inForm (v2.6.0)<br>phenoptr Reports (v0.3.2)<br><br>Flow Cytometry:<br>FlowJo (v10.8.0)<br><br>NGS:<br>BWA mem (v0.7.15)<br>SAMtools mpileup (v 1.16.1)<br>VarScan2 (v2.4.3)<br>ANNOVAR (v2018-04-16 - https://annovar.openbioinformatics.org/en/latest/)<br>MSIsensor2 (v0.1 - https://github.com/niu-280 lab/msisensor2)<br><br>RNA-Seq:

R (v4.1.2)
STAR (v2.6.1)
edgeR (v3.40.1)
Gencode (v29)
Zenodo (v3.1.8 - https://github.com/bioinfo-pf-curie/RNA-seq, DOI : 10.5281/zenodo.7446922)
limma (v3.54.0)
fgsea (v1.24.0)
GSVA (v1.46.0)
MSigDB (v7.5.1)
ggplot2 (v3.4.0)
ggrepel (v0.9.2)
ComplexHeatmap (v2.14.0)
Maftools (v4)

Other:
Graphpad prism (v9.5.0)

For manuscripts utilizing custom algorithms or software that are central to the research but not yet described in published literature, software must be made available to editors and reviewers. We strongly encourage code deposition in a community repository (e.g. GitHub). See the Nature Portfolio guidelines for submitting code & software for further information.

Data

Policy information about availability of data

All manuscripts must include a data availability statement. This statement should provide the following information, where applicable:

- Accession codes, unique identifiers, or web links for publicly available datasets
- A description of any restrictions on data availability
- For clinical datasets or third party data, please ensure that the statement adheres to our policy

Data Availability Statement included in the manuscript:
The raw and processed data from both targeted DNA and RNA sequencing have been deposited in the EGA database under accession code EGAS00001007297. The data are available under restricted access after review of the Data Access Committee (DAC) because these are human data generated from medical research. Request for data access will be referred directly to our DAC at data.request@curie.fr (https://ega-archive.org/dacs/EGAC00001003266). Individual, de-identified, participant sequencing and clinical data will be accessible for research purpose only as specified in the data access policy. After review of the request, data access decision will be passed to the EGA database within four weeks, and an access account will then be granted. In case of publication, the data will be available for 2 years from the date of last publication. If there is no use of the data for a period of 2 years, the requester has to delete the data. The remaining data are available within the Article, Supplementary Information or Source Data file. Source data are provided with this paper.

Human research participants

Policy information about studies involving human research participants and Sex and Gender in Research.

Reporting on sex and gender	The findings of this paper apply to female individuals only (study on cervical cancer).
Population characteristics	Population characteristics are carefully detailed in Table 1.
Recruitment	Between November 2017 and July 2020, 21 patients with locally-advanced cervical cancer were were screened for eligibility and 16 patients were finally included in the study at the Institut Curie, based on the inclusion criteria of the clinical trial. The patients were discussed at the Tumor Board to avoid any self-selection bias and were included sequentially.
Ethics oversight	The Clermont-Ferrand ethic committee (CPP Sud-Est VI, AU 1316) approved the trial, which was conducted in accordance with the Declaration of Helsinki and the national regulatory requirements.

Note that full information on the approval of the study protocol must also be provided in the manuscript.

Field-specific reporting

Please select the one below that is the best fit for your research. If you are not sure, read the appropriate sections before making your selection.

☒ Life sciences ☐ Behavioural & social sciences ☐ Ecological, evolutionary & environmental sciences

For a reference copy of the document with all sections, see nature.com/documents/nr-reporting-summary-flat.pdf

Life sciences study design

All studies must disclose on these points even when the disclosure is negative.

Sample size	A total of 16 eligible patients were included in the trial and in the translational analyses.
Data exclusions	Due to poor samples quality, one patient with partial response was excluded from Flow Cytometry, Multiplex-IF and RNA-seq analyses.
Replication	All data point represent individual biological replicates. In our study, we did not perform technical replicates.
Randomization	NiCOL is a phase 1 clinical trial designed to determine safety, tolerance, and explore immune correlates of response to the PD-1 blocking antibody, nivolumab, with and following concurrent chemoradiotherapy. The design of the study did not require randomization as it was a safety/toxicity study.
Blinding	No blinding was performed to allow the assessment of the safety and proximity profiles of the combination treatment.

Reporting for specific materials, systems and methods

We require information from authors about some types of materials, experimental systems and methods used in many studies. Here, indicate whether each material, system or method listed is relevant to your study. If you are not sure if a list item applies to your research, read the appropriate section before selecting a response.

Materials & experimental systems

n/a	Involved in the study	
☐	☒	Antibodies
☒	☐	Eukaryotic cell lines
☒	☐	Palaeontology and archaeology
☒	☐	Animals and other organisms
☐	☒	Clinical data
☒	☐	Dual use research of concern

Methods

n/a	Involved in the study	
☒	☐	ChIP-seq
☐	☒	Flow cytometry
☒	☐	MRI-based neuroimaging

Antibodies

Antibodies used

Multiplex Immunohistochemistry antibodies:
CD3 (polyclonal, cat. A0452, Agilent, 1:400)
CD8 (clone C8/144B, cat. M7103, Agilent, 1:100)
CD11c (clone 2F1C10, cat. 60258-1-Ig, Protein tech, 1:10 000)
CD28 (clone EPR22076, cat. ab243228, Abcam, 1:500)
CD86 (clone EP1158-37, cat. ab269587, Abcam, 1:50)
Cytokeratin (clone AE1/AE3, cat. M3515, Agilent, 1:200)
FOXP3 (clone 236A/E7, cat. ab20034, Abcam, 1:200)
Granzyme B (clone GrB-7, cat. M7235, Agilent, 1:100)
Ki67 (clone MIB-1, cat. Dako M7240, Agilent, 1:1000)
PD-1 (clone EPR4877(2), cat. ab137132, Abcam, 1:500
PD-L1 (clone ZR3, cat. Z2002RL, Diagomics, 1:600)

Flow Cytometry Antibodies (mouse anti-human):
CD1c (clone L161, BV421, cat. 331526, Biolegend, 1:60)
CD3 (clone UCHT1, APC-Cy7, cat. 300426, Biolegend, 1:50)
CD3 (clone UCHT1, AF700, cat. 300424, Biolegend, 1:50)
CD4 (clone OKT4, BV650, cat. 317436, Biolegend, 1:100)
CD8 (clone 3B5, PE-Texas Red, cat. MHCD0817, ThermoFisher Scientific, 1:80)
CD11c (clone B-ly6, BUV395, cat. 563787, BD, 1:50)
CD14 (clone M5E2, AF488, cat. 557700, BD, 1:50)
CD16 (clone 3G8, BUV737, cat. 564434, BD, 1:100)
CD19 (clone HIB19, AF700, cat. 302226, Biolegend, 1:50)
CD25 (clone BC96, BV605, cat. 302623, Biolegend, 1:40)
CD27 (clone M-T271, PerCP-Cy5.5, cat.356408, Biolegend, 1:100)
CD45 (clone 2D1, APC-Cy7, cat. 557833, BD, 1:50)
CD45RA (clone HI100, BV786, cat. 563870, BD, 1:120)
CD56 (clone B159, AF700, cat. 557919, BD, 1:100)
CD56 (clone NCAM16.2, BUV395, cat. 563554, BD, 1:100)
CD123 (clone 6H6, AF647, cat. 306024, Biolegend, 1:100)

CD127 (clone A01905, AF488, cat. 351314, Biolegend, 1:80)
CD141 (clone M80, BV786, cat. 344116, Biolegend, 1:50)
CD141 (clone M80, PerCP-Cy5.5, cat. 344112, Biolegend, 1:25)
CD163 (clone RM3/1, AF647, cat. 326508, Biolegend, 1:80)
CD206 (clone 19.2, PE-CF594, cat. 564063, BD, 1:50)
EpCAM (clone 9C4, BV785, cat. 324238, Biolegend, 1:100)
HLA-DR (clone L243, BV650, cat. 307650, Biolegend, 1:120)
ICOS (clone DX29, BUV737, cat. 564778, BD, 1:25)
ICOS-L (clone 2D3, PE-Cy7, cat. 309410, Biolegend, 1:25)
OX40 (clone Ber-ACT35, PE, cat. 350004, Biolegend, 1:25)
Ki67 (clone Ki67, BV421, cat. 350506, Biolegend, 1:50)
PD-L1 (clone MIH1, BV421, cat. 563738, BD, 1:30)
PD-L1 (clone MIH1, PerCP-eFluor710, cat. 46-5983-42, eBioscience, 1:50)
PD-1 (clone EH12.2H7, AF647, cat. 329910, Biolegend, 1:50)
IgG4 (secondary ab) (clone HP-6025, Biotin, cat. B3648, Sigma, 1:100)
Streptavidin (AF647, cat. 405237, Biolegend, 1:200)

Isotype controls:
Mouse IgG1, κ (OX40 Isotype - clone MPC-11, PE, cat. 400114, Biolegend)
Mouse IgG1, κ (ICOS Isotype - clone X40, BUV737, cat. 564299, BD)
Mouse IgG2b, κ (ICOS-L Isotype - clone MOPC-11, PE-Cy7, cat. 400326, Biolegend)
Mouse IgG1, κ (PD-L1 Isotype - clone P3.6.2.8.1, PerCP-eFluor710, cat. 25-4714-42, eBioscience)
Mouse IgG1, κ (PD-L1 Isotype) MOPC-21 BV421 400157 Biolegend)

Validation

The multiplex-IHC antibodies were validated on the following tissues:
CD3 : Tonsil
CD8 : Tonsil
CD11c : Tonsil
CD28 : Tonsil
CD86 : Tonsil
Cytokeratin : Breast
FOXP3 : Tonsil
Granzyme B : Tonsil
Ki67 : Tonsil
PD-1 : Tonsil
PD-L1 : Tonsil /Placenta

The flow cytometry antibodies were validated on the following targets:
CD1c : PBMCs
CD3 : PBMCs/Lymphocytes
CD3 : PBMCs/Lymphocytes
CD4 : PBMCs/Lymphocytes
CD8 : PBMCs/Lymphocytes
CD11c : PBMCs
CD14 : PBMCs/Monocytes
CD16 : PBMCs
CD19 : PBMCs/Lymphocytes
CD25 : 3-days PHA-stimulated PBMCs/Lymphocytes
CD27 : PBMCs
CD45 : PBMCs/Lymphocytes
CD45RA : PBMCs/Lymphocytes
CD56 : PBMCs/Lymphocytes
CD56 : PBMCs/Lymphocytes
CD123 : PBMCs
CD127 : PBMCs/Lymphocytes (CD3+)
CD141 : overnight LPS-stimulated PBMCs/Monocytes
CD141 : overnight LPS-stimulated PBMCs/Monocytes
CD163 : overnight IL-10-stimulated PBMCs/Monocytes
CD206 : 3-days GM-CSF-stimulated PBMCs/Monocytes
EpCAM : HT29 cell line
HLA-DR : PBMCs/Monocytes
ICOS : 3-days PHA-stimulated PBMCs/Lymphocytes
ICOS-L : Burkitt's lymphoma cell line Daud
OX40 : 3-days PHA-stimulated PBMCs/Lymphocytes
Ki67 : 3-days PHA-stimulated PBMCs/Lymphocytes
PD-L1 : 3-days PHA-stimulated PBMCs/Lymphocytes
PD-L1 : 3-days PHA-stimulated PBMCs/Lymphocytes
PD-1 : PBMCs/Lymphocytes (CD3+)

Clinical data

Policy information about clinical studies

All manuscripts should comply with the ICMJE guidelines for publication of clinical research and a completed CONSORT checklist must be included with all submissions.

Clinical trial registration	NCT03298893
Study protocol	The NiCOL trial (NCT03298893) is an open-label, single-arm, phase-I, dose-confirmation, multicenter trial aiming to determine safety and tolerance, and immune correlates of concurrent and maintenance nivolumab plus Chemo-Radiation therapy (CRT) in locally-advanced cervical cancer (LACC) patients. Inclusion criteria included: immunotherapy-naïve adult patients with histologically confirmed cervical adenocarcinoma or cervical squamous, Federation of Gynecology and Obstetrics (FIGO) 2018 stages IB3-IVA, with an indication for curative intent cisplatin-based CRT.<br>The disease had to be amenable to biopsy. Exclusion criteria included: distant metastatic disease, prior history of radiotherapy, systemic antineoplastic treatment or clinically significant comorbidities. The Clermont-Ferrand ethic committee (CPP Sud-Est VI, AU 1316) approved the trial, which was conducted in accordance with the Declaration of Helsinki and the national regulatory requirements. All patients signed a written informed consent.
Data collection	Biological samples were collected during the recruitment phase between November 2017 and July 2020 at the institutional biobank of Institut Curie.
Outcomes	The primary endpoint was the incidence of dose-limiting toxicities (DLT) within 11 weeks after the initiation of treatment, corresponding to the first six cycles of nivolumab. DLT were graded according to the Common Terminology Criteria for Adverse Events (CTCAE), version 4.03.<br>Secondary endpoints included overall response rate (ORR), progression-free survival (PFS) and treatment tolerance profile. ORR was radiologically defined according to RECIST 1.1 criteria based on thoracic-abdominal-pelvic computed tomography, completed with pelvic MRI and 18F-FDG PET-CT evaluations. ORR was defined as the proportion of all subjects whose best response was either a complete response (CR) or a partial response (PR), as assessed using RECIST 1.1 criteria. PFS was defined as the duration from start of the treatment to disease progression, date of last follow-up or death, regardless of cause of death. PFS was estimated by the Kaplan-Meier method.<br>Exploratory analyses included immunological and molecular correlates of response to therapy.

Flow Cytometry

Plots

Confirm that:

- [x] The axis labels state the marker and fluorochrome used (e.g. CD4-FITC).
- [x] The axis scales are clearly visible. Include numbers along axes only for bottom left plot of group (a 'group' is an analysis of identical markers).
- [x] All plots are contour plots with outliers or pseudocolor plots.
- [x] A numerical value for number of cells or percentage (with statistics) is provided.

Methodology

Sample preparation	Following enzymatic dissociation (detailed in the M&M section of the manuscript), cells were stained with LIVE/DEAD™ Fixable Aqua Dead Cell Stain Kit (TermoFisher Scientific, Cat#L34957), according to the manufacturer's instruction, for dead cell exclusions.<br>For both tumor and peripheral blood myeloid panels, a 10min pre-incubation at +4ºC with 1:25 PBS-diluted Fc receptor binding inhibitor (eBioscience, Cat#14-9161-73) was performed, before direct addition of the antibody mixes.<br>For surface staining, cells were incubated with the primary antibody mix (in FACS buffer) for 20min at +4ºC in the dark, washed, resuspended in FACS buffer and stored at +4ºC covered in aluminium foil, until analysis.<br>For intracellular staining of Ki67, surface stained cells were fixed and and permeabilized with the FOXP3 fixation-permeabilization kit (Thermo Fischer, Cat#00-214 5521-00) according to the manufacturer's instruction. Then, cells were intracellularly stained for 20min at +4ºC in the dark, washed, resuspended in FACS buffer and stored at +4ºC covered in aluminium foil, until analysis.
Instrument	Data were acquired on a BD LSR Fortessa.
Software	FlowJo (version 10.8.0)
Cell population abundance	Cell sorting was not performed in this study.
Gating strategy	Cells debris were excluded with FSC-A/SSC-A gating. Cell singlets were gated according to FSC-A/FSC-W and SSC-A/SSC-W parameters. Gating on Aqua LIVE/DEAD negative cells, and according to the panel of antibody used, the cell population of interest are identified (see Supplementary Figures 2 and 3). To properly set-up the voltages and to determine appropriate gates, isotype antibodies were used as controls.

- [x] Tick this box to confirm that a figure exemplifying the gating strategy is provided in the Supplementary Information.
